# Symmetry-Breaking Charge-Separation in a Subphthalocyanine Dimer Resolved by Two-Dimensional Electronic Spectroscopy

**DOI:** 10.1021/acs.jpcc.4c07588

**Published:** 2025-01-03

**Authors:** Giovanni Bressan, Isabelle Chambrier, Andrew N. Cammidge, Stephen R. Meech

**Affiliations:** School of Chemistry, University of East Anglia, Norwich NR4 7TJ, U.K.

## Abstract

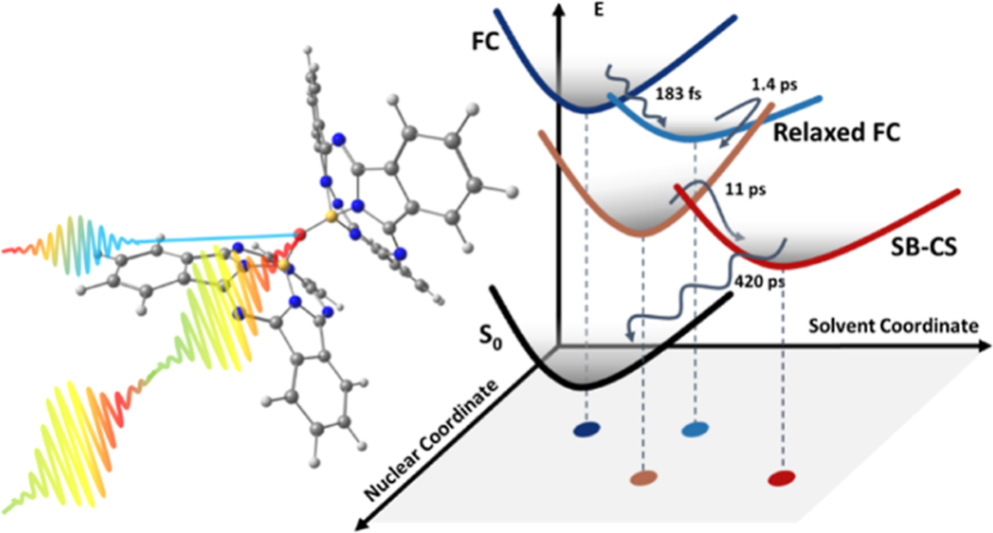

Understanding the role of structural and environmental dynamics in the excited state properties of strongly coupled chromophores is of paramount importance in molecular photonics. Ultrafast, coherent, and multidimensional spectroscopies have been utilized to investigate such dynamics in the simplest model system, the molecular dimer. Here, we present a half-broadband two-dimensional electronic spectroscopy (HB2DES) study of the previously reported ultrafast symmetry-breaking charge separation (SB-CS) in the subphthalocyanine oxo-bridged homodimer μ-OSubPc_2_. Electronic structure calculations and 2D cross-peaks reveal the dimer’s excitonic structure, while ultrafast evolution of the multidimensional spectra unveils subtle features of structural relaxation, solvation dynamics, and inhomogeneous broadening in the SB-CS. Analysis of coherently excited vibrational motions reveals dimer-specific low-frequency Raman active modes coupled to higher-frequency vibrations localized on the SubPc cores. Finally, beatmap amplitude distributions characteristic of excitonic dimers with multiple bright states are reported and analyzed.

## Introduction

Strongly coupled chromophores play a central role in many photonic materials. The model system for understanding the photophysics of interchromophore coupling is the molecular homodimer, which exhibits multiple excited state relaxation pathways including excimer formation, singlet fission, and symmetry-breaking charge separation (SB-CS).^1−6^ Understanding how these processes are coupled to dimer geometry, intradimer structural dynamics, and the external medium is of critical importance, and has been widely studied by ultrafast spectroscopy including two-dimensional electronic spectroscopy (2DES).^3,7−11^

**Figure 1:**
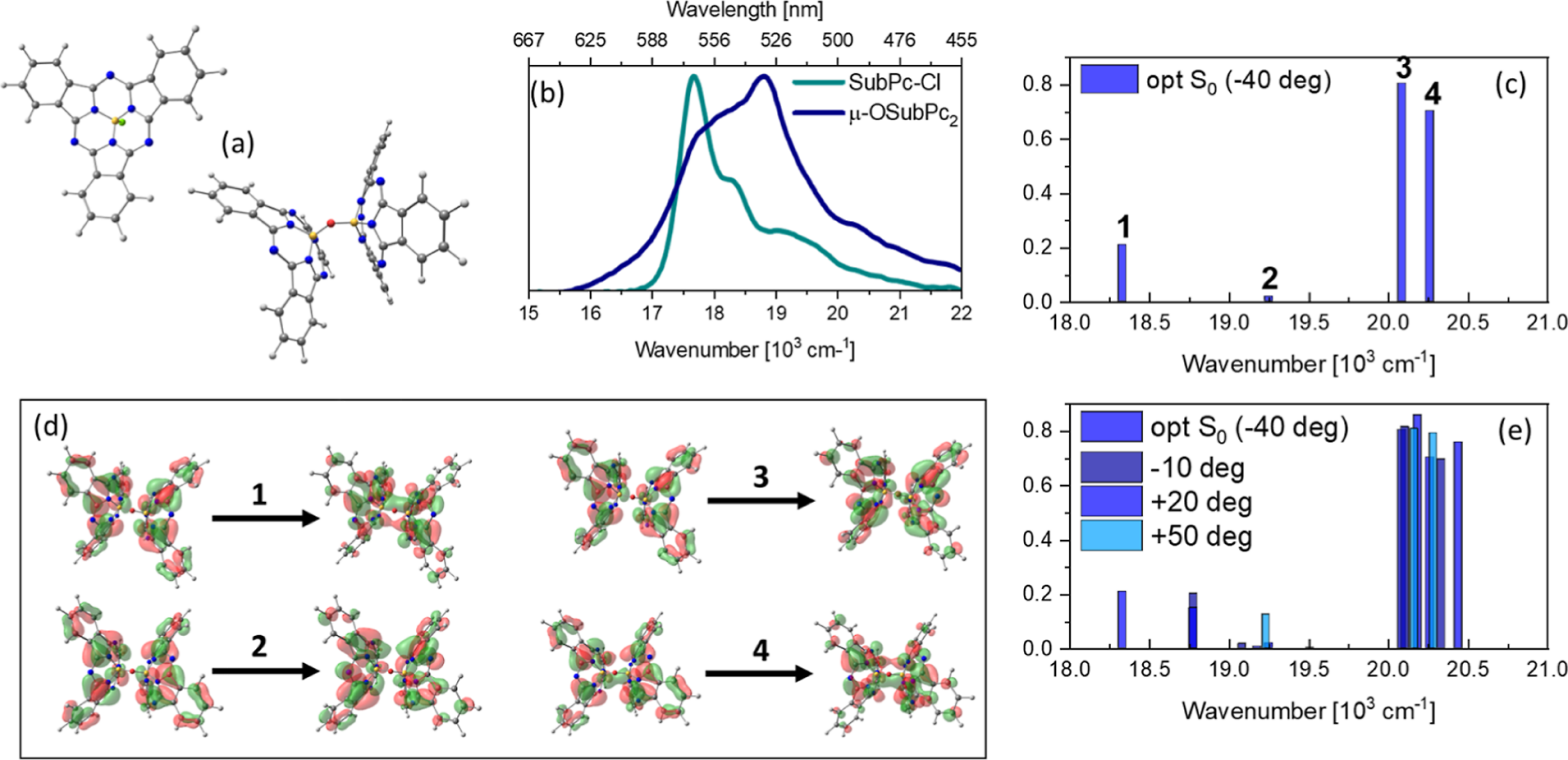
(a) Optimized ground state (S_0_) geometries of SubPc-Cl and μ-OSubPc_2_. (b) Normalized steady-state absorption spectra of SubPc-Cl (teal) and μ-OSubPc_2_ (blue). (c) Stick spectrum showing the excitonic transitions of μ-OSubPc_2_ calculated by TD-DFT at the S_0_ equilibrium conformation. (d) Natural transition orbitals (NTOs) of the four excitonic transitions shown in (c). (e) Excitonic transitions calculated by TD-DFT as a function of consecutive 30-degree rotations around a N–B–O–B dihedral of the S_0_-optimized μ-OSubPc_2_ structure shown in (a).

In this work, we report a half-broadband two-dimensional electronic spectroscopy (HB2DES) study of the subphthalocyanine (SubPc) oxo bridged homodimer, μ-OSubPc_2_ (Figure 1a), in a polar N–N dimethylformamide (DMF) solvent (ε = 37), in which SB-CS was previously reported in a transient absorption study.^5^ SubPcs have great potential in photonics because of their strong absorption in the visible region and resistance to aggregation due to the nonplanar structure (Figure 1a).^12−15^ Both homo- and heterodimers of SubPc have been synthesized. Their electronic spectra show absorption and emission features characteristic of excitonic coupling, and their excited states dynamics encompass energy transfer, charge separation, and intersystem crossing (ISC).^16−20^ The μ-OSubPc_2_ dimer has been studied previously and was shown to display the characteristic absorption of a coupled dimer and solvent-dependent excimer formation and/or SB-CS on an ultrafast time scale.^5^ In addition to its potential photonics application, μ-OSubPc_2_ (Figure 1a) is an unusual example of a cofacial non-coplanar strongly interacting homodimer, such as is found in the special pair of the photosynthetic reaction center,^21^ which also supports SB-CS. The present application of HB2DES to μ-OSubPc_2_ in polar DMF reveals the earliest stages of the ultrafast evolution from the Franck–Condon (FC) region of the excited dimer toward the charge-separated state and probes the sub-ps coherent dynamics arising from coupled structural and excitonic degrees of freedom.

## Experimental Methods

Steady-state UV–vis spectra were recorded in static 1 mm fused silica cells (Starna) at 450 mOD on a PerkinElmer Lambda XLS benchtop instrument. Steady-state emission spectra were recorded in static 10 mm fused silica cells (Hellma) at 100 mOD at an excitation wavelength of 19,000 cm^–1^. The fluorimeter is an Edinburgh Instruments FS5.

HB2DES data were acquired on 450 mOD solutions (1 mm optical path static cell) of μ-OSubPc_2_ (synthesized and purified as previously described in Roy et al.^5^) and SubPc-Cl (synthesized and purified as previously described by Claessens et al.^22^ and in the Supporting Information of Roy et al.^5^) in DMF. Samples and solvents were used as received. The home-built half broadband 2D experimental setup has been previously described in detail.^23^ 500 mW from the output of a Ti/Sa regenerative amplifier (Spitfire Ace, Spectra-Physics) running at a 1 kHz repetition rate and centered at 800 nm drives a commercial noncollinear optical parametric amplifier (NOPA, Topas White, Light Conversion). The NOPA output (∼700 nJ energy per pulse pair) is precompressed by a commercial folded grism compressor (Fastlite) to achieve close to transform limited pulses at the sample position. Downstream, a pair of pump pulses with programmable time delay (coherence time τ) and relative carrier envelope phase is created in a commercial acousto-optical programmable dispersive filter (AOPDF, Dazzler, Fastlite). The coherence time is scanned shot-to-shot from 0 to 95 fs in 0.792 fs steps. A three-frame phase-cycling scheme is used to collect the real and imaginary parts of the rephasing and non-rephasing responses, which are then combined to yield absorptive 2D spectra.^24^ Each 2D spectrum is averaged over 180 laser shots per value of τ. The waiting time *T* is introduced by scanning the pump pair against the probe by a retroreflector mounted on a mechanical delay stage (Physik Instrumente), in 10 fs steps from 0 to 1200 fs to record vibrationally coherent dynamics or at increasing *T* steps between 0 and 100 ps for the population measurements. The probe pulse (white light continuum, WLC) is generated by focusing ∼1 mW of the 800 nm regenerative amplifier output into a 3 mm static sapphire plate and spans 13,500–23,000 cm^–1^. The WLC is passed in a compressor made of two pairs of dispersive mirrors at 5 and 19° angles of optical incidence (PC 1332, Ultrafast Innovations), split by a 50:50 beamsplitter and crossed at ∼4° with the collinear pump pair at the sample position. Pump(s) and probe spot sizes are 80 and 160 μm, respectively. The signal is recollimated after the sample and the signal and reference are focused into a home-built dual channel prism-based spectrometer to be acquired shot-to-shot by a pair of 1024 pixels CCD detectors (Stresing) synchronized to the AOPDF. The signal is referenced using an active noise reduction method proposed by Feng et al.^25^ The instrument response function (ca. 50 fs; see Supporting Information Figure S2) is measured by spectrally resolving the cross-correlation between NOPA and WLC pulses in neat toluene. The relative polarization between pumps and probe was set at the magic angle for all measurements.

## Results and Discussion

### Steady-State Spectroscopy

The steady-state electronic absorption spectra of the monomer SubPc-Cl and μ-OSubPc_2_ in DMF are shown in Figure 1b and are essentially the same as previously reported in toluene.^5,17^ Thus, the absorption spectra of SubPc-Cl and μ-OSubPc_2_ are only weakly influenced by medium polarity. The monomer displays a strong (∼4.5 × 10^4^ M^–1^ cm^–1^) absorption in the blue-near UV, B- (or Soret) band region, and a more intense (∼7 × 10^4^ M^–1^ cm^–1^) and red-shifted Q-band at ∼17,500 cm^–1^, which is the focus of this study. The Soret and Q-bands arise from interactions between the transition dipole moments (TDMs) of a pair of HOMO–LUMO transitions, as originally described for porphyrins by Gouterman.^26^ Both Soret- and Q-band transitions are doubly degenerate in SubPc-Cl due to its C_3v_ symmetry (Figure 1a).^27^

The electronic absorption of μ-OSubPc_2_ in DMF shows an intense peak at 19,200 cm^–1^, a broad shoulder at 17,600 cm^–1^, and a weaker “tail” extending to 15,700 cm^–1^. To a first approximation, this dimer spectrum can be rationalized by Kasha’s dipole coupling model,^28^ which predicts a doublet of states, red- and blue-shifted from the transition energy of the uncoupled chromophore, arising from in- and out-of phase interactions between the TDM of each chromophore. Hence, these are labeled as |+⟩ and |−⟩ states, respectively. The magnitude of such shifts is given by the strength of the excitonic coupling *J*, which depends on long- (Coulomb) and short- (charge transfer) range forces between chromophores.^29^ The oscillator strengths of |+⟩ and |-⟩ depend on the relative orientations of the coupled TDMs. An angle of 0° (90°) between TDMs makes the transition to the upper (lower) exciton state forbidden, causing a red-(blue-) shift of the dimer absorption with respect to the monomer. The μ-OSubPc_2_ has an “intermediate” geometry (from its DFT optimized S_0_ geometry, see Figure 1a, the angle between the planes containing the isoindole nitrogen atoms is 41.6°, in good agreement with the 39-degree angle measured in the crystalline phase^27^). Consequently, all excitonic states, split by 2*J*, carry an oscillator strength. Because the degeneracy between the pair of Q-band transitions in the SubPc-Cl is lifted in μ-OSubPc_2_, each TDM couples to both transitions of the neighboring chromophore, to yield four nondegenerate excitonic states with different TDMs, which are both blue- and red-shifted from the SubPc-Cl absorption. Qualitatively, this model is consistent with the observed red and blue shifts seen in the dimer spectrum relative to the monomer (Figure 1b).

To investigate the spectrum in more detail, the electronic transitions of SubPc-Cl and μ-OSubPc_2_ were calculated by TD-DFT at their optimized S_0_ geometries (Figure 1b) at the ωb97xd/dgdzvp level of theory, including a polarizable continuum model appropriate for DMF solvent. All the calculations were conducted in Gaussian 16.^30^ In the SubPc-Cl monomer, the measured Q-band absorption corresponds to the calculated S_1_ ← S_0_ and S_2_ ← S_0_ transitions (Figure S1), which are degenerate with identical oscillator strengths. The calculated vertical excitation energy is overestimated by ∼1700 cm^–1^, consistent with earlier observations for this level of theory.^31^

The calculated electronic stick spectrum of μ-OSubPc_2_ reported in Figure 1c shows four transitions in the Q-band region between 18,300 and 20,300 cm^–1^, again overestimating the energies of the experimental spectrum, in this case by ∼1500 cm^–1^. The calculated TDMs differ by a factor of ∼40 and split into two pairs centered at 18,750 and 20,200 cm^–1^, respectively; a similar result was reported for the rigid nonparallel substituted porphyrin dimers by Roy et al.^31^ and for BODIPY aggregates by Ghosh et al.^32^ The highest occupied/lowest unoccupied NTOs of the four excitonic transitions are shown in Figure 1d and show how the largest values of *J* (transitions 1 and 4) involve substantial charge redistribution around the B–O–B bridge, while transitions 2 and 3 are more localized on the SubPc moieties. These NTOs are similar in pairs, as they represent transitions to excitonic states deriving from the coupling of two identical SubPc units, each with a pair of orthogonal Q-band TDMs (Figure S1). Further, because in μ-OSubPc_2_, nearly barrierless (∼0.5 kJ/mol) rotation around the B–O bonds is expected at room temperature,^5^ electronic spectra were calculated as a function of such rotation, in 30-degree steps, (0(120), 30, 60, 90). The results are shown in Figure 1e. Rotation affects both the TDMs and vertical excitation energies, leading to the emergence of a pair of weak and strong excitonic “bands” red- and blue-shifted with respect to the SubPc-Cl Q-band transitions (see Supporting Information Figure S1). It is worth noting that this simplified model for the excitonic transitions of μ-OSubPc_2_ does not include vibronic effects or Herzberg–Teller couplings, which might be relevant in SubPcs, as they are known to play a significant role in the absorption spectra of structurally related porphyrins.^33^ Broadening arising from such phenomena is thus not included. Nevertheless, the calculation allows us to assign the steady-state absorption spectrum of μ-OSubPc_2_ in DMF to an inhomogeneously broadened ground state distribution of rotamers, whose four excitonic transition energies and TDMs are a function of the dihedral angle between the TDMs of each SubPc unit.

### Population Dynamics

**Figure 2:**
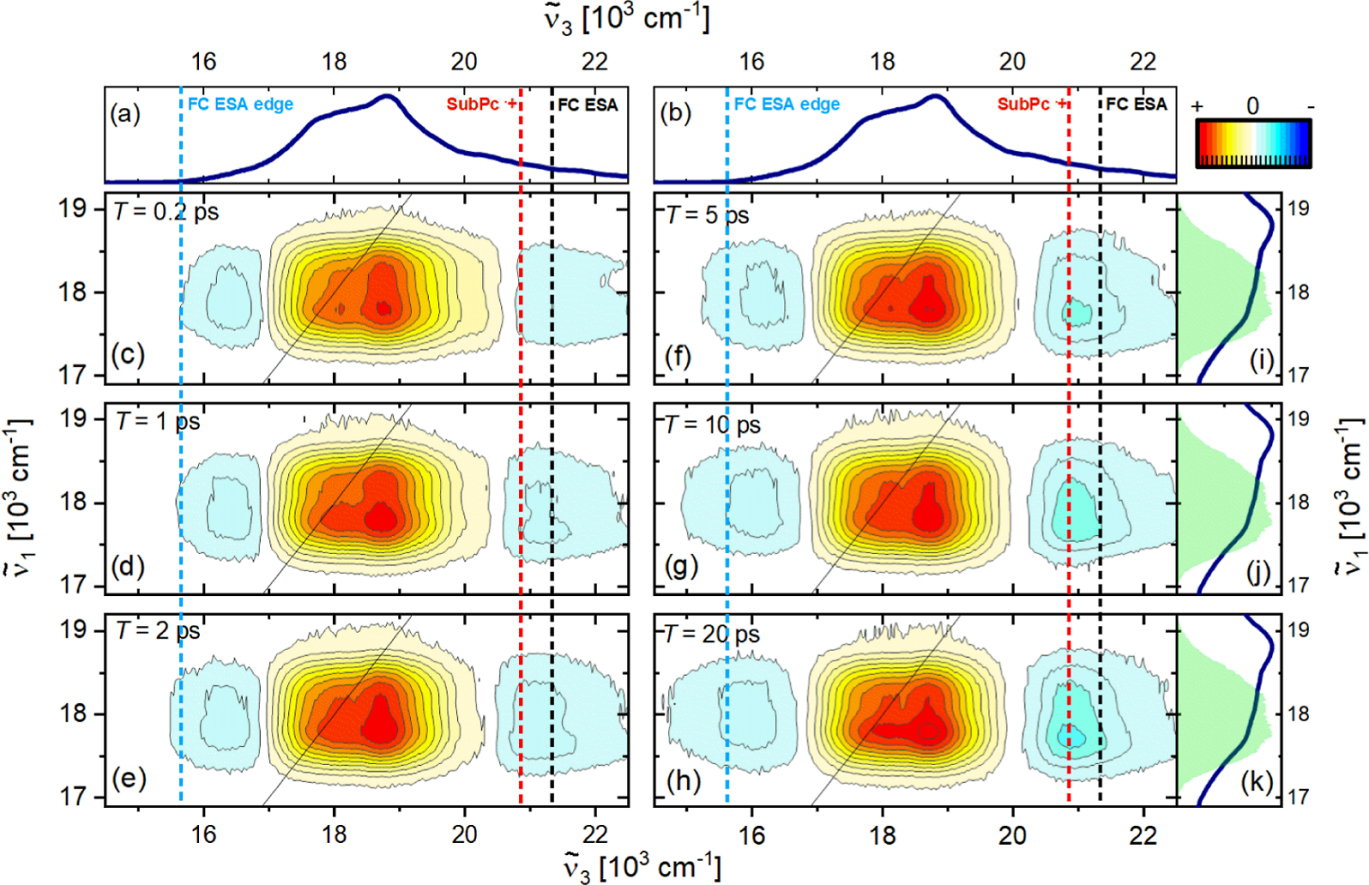
(a,b) Normalized steady-state absorption of μ-OSubPc_2_ in DMF. (i–k) Pump spectrum used for the HB2DES measurements and normalized steady-state absorption of μ-OSubPc_2_ in DMF are shown as shaded green and solid blue lines, respectively. The absorptive 2D spectra of μ-OSubPc_2_ at *T* = 0.2, 1, 2, 5, 10, 20 ps are shown in (c–h), respectively. The intensity is given by 21 contour lines; positive signals are shown in yellow-orange-red, and negative signals are shown in blue. All spectra are normalized to the positive GSB amplitude at *T* = 0.2 ps. As a guide to the eye, vertical light blue, red, and black dashes indicate the edge of the low energy excited state absorption (ESA) and the absorption maxima of the SubPc^•+^ and of the high-energy ESA, respectively at *T* = 0.2 ps.

Next, we apply HB2DES to probe excitonic coupling in μ-OSubPc_2_ allowing us to further characterize the previously reported ultrafast SB-CS dynamics in polar DMF with improved spectral and temporal resolution. HB2DES is a χ^(3)^ method relying on a three-pulse sequence in a partially collinear geometry and the absorptive HB2D electronic spectra of SubPc-Cl and μ-OSubPc_2_ in DMF can be thought of as excitation frequency () resolved broadband transient absorption spectra. The samples were excited by a pair of ∼30 fs pulses separated by the coherence time (τ) and centered at  = 18,000 cm^–1^ (shaded green in Figure 2). After a waiting time *T*, the samples were probed by a compressed WLC.

The HB2DES of SubPc-Cl at *T* = 0.2 ps is reported in the Supporting Information (Figure S3) and is in excellent agreement with fsTA and one-color 2DES literature data in toluene, indicating weak dependence of the monomer photophysics on the polarity of the solvent environment.^5,17^ Absorptive HB2DES of μ-OSubPc_2_ in DMF were measured between *T* = 0 and 100 ps and selected values of *T* between 0.2 and 20 ps are shown in Figure 2, where normalized steady-state absorption and NOPA pump spectra are reproduced to facilitate interpretation. At *T* = 0.2 ps, the absorptive spectrum of μ-OSubPc_2_ in DMF (Figure 2c) presents a broad positive feature matching the steady-state absorption, hence assigned to ground state bleach (GSB). There are two negative contributions centered at  = 16,300 and 21,300 cm^–1^, with the latter extending toward the blue edge of the WLC probe, which are assigned to S_*n*_ ← S_1_ ESA, in agreement with published fsTA.^5^ To highlight spectral evolution, the detection wavenumbers corresponding to the ESA maximum (at *T* = 0.2 ps) at  = 21,300 cm^–1^ and to the red-edge of the ESA at  = 16,300 cm^–1^ are highlighted by vertical black and light blue dashed lines, respectively. Further, a vertical red dashed line at  = 20,800 cm^–1^ indicates the maximum of the SubPc^•+^ absorption, which is expected to be a characteristic feature of SB-CS in this dimer.^15^

The cross peak at  17,600,  18,800 cm^–1^, (|+⟩,|-⟩) is present at the earliest waiting times (absorptive HB2DES at *T*s between 0 and 125 fs are shown in Figure S4) and is absent in the HB2DES of SubPc-Cl. This cross-peak provides a strong indication of excitonic coupling in μ-OSubPc_2_.^34,35^ Due to the limited spectral coverage of the pump pulses, the diagonal peak corresponding to the upper exciton band |−⟩ at  18,800 cm^–1^ is not resolved. Similarly, the cross-peak at  18,800,  17,600 cm^–1^ (|−⟩,|+⟩) is filtered out by the finite bandwidth of the excitation pulses. It is worth highlighting that one-color 2DES experiments cannot resolve excitonic cross-peaks unless the laser bandwidth spans (at least) a pair of coupled states. Conversely, in HB2DES, even if the pump pulses span only one excitonic state, the broadband WLC probe will detect GSB cross-peaks at  positions corresponding to every excitonic level coupled to the transition excited by the pump.

The ps evolution of the HB2DES of μ-OSubPc_2_ is dominated by a ∼500 cm^–1^ red-shift and growth in amplitude of the negative signal at  = 20,000–22,000 cm^–1^, (see red dash line), which also causes a narrowing of the GSB. Such a rise is coupled to the reshaping and broadening toward the red edge of  of the weaker negative band below 17,000 cm^–1^, as revealed by following the light blue dashed line. Evolution of the negative bands is due to the onset of SubPc radical anion (21,000 cm^–1^) and cation (14,000 and 19,200 cm^–1^) absorption bands, whose formation is a distinctive feature of SB-CS. Further, the formation of a CS state is proven by the stimulated emission (SE) being quenched within a few picoseconds from photoexcitation. The ps dynamics and spectral evolution retrieved by HB2DES are in excellent agreement with the spectral evolution due to SB-CS observed by fsTA.^5^ Closer inspection of the region below 17,000 cm^–1^ reveals sub-200 fs formation of a weak positive signal centered at  = 15,000 cm^–1^ (this feature is better resolved in the HB2DES integrals over  reported in the Supporting Information, Figure S5), which disappears within ∼5 ps. This is assigned to the SE. The excited state dynamics are accompanied by elongation toward lower  of the GSB cross-peak in the  = 17,500–17,700 cm^–1^;  178,000–18,800 cm^–1^ region.

**Figure 3:**
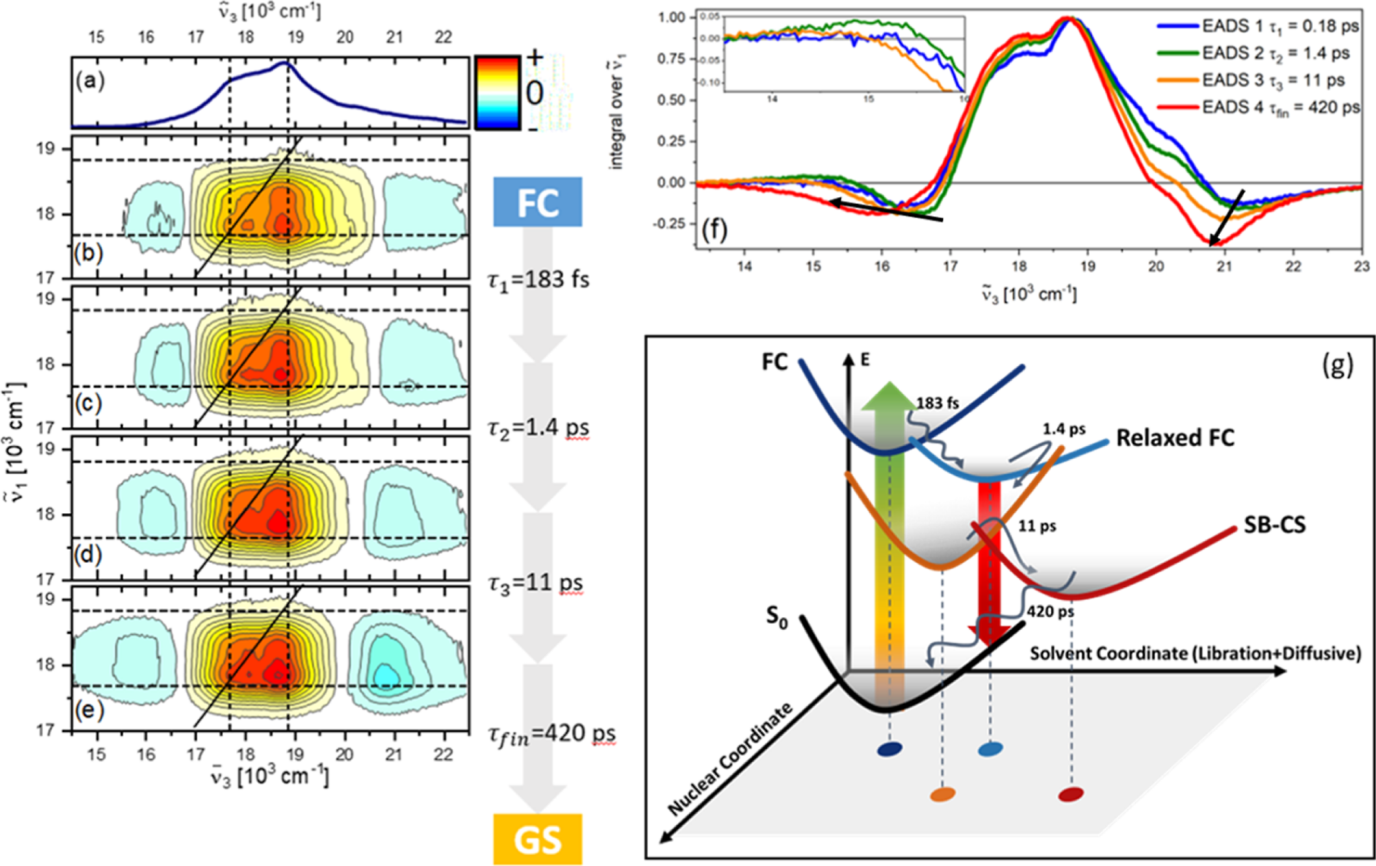
(a) Normalized steady-state absorption of μ-OSubPc_2_ in DMF. (b–e) Normalized 2D evolution-associated difference spectra (EADS) obtained by global fitting of the absorptive HB2DES of μ-OSubPc_2_ in DMF. The intensity is given by 21 contour lines; positive signals are shown in yellow-orange-red, and negative signals are shown in blue. Dashes indicate the position of the |+⟩ and |−⟩ excitonic states. (f) Integrals of the normalized EADS over the excitation axis. Superimposed arrows highlight the redshift, rise time, and reshaping of the negative bands due to formation of the SubPc radical pair, which is the distinctive feature of the SB-CS reaction. The inset highlights the weak SE signal in EADS2. (g) Potential energy surface (PES) diagram showing the evolution from Franck–Condon to SB-CS states with recovered time constants of each state. Solid upward and downward arrows represent absorption and SE, respectively. Colored dots indicate positions of the PES minima on the plane defined by nuclear and solvation coordinates.

The dynamical evolution of the HB2DES data is quantified by global fitting analysis, assuming sequential evolution through a series of first-order steps with increasing time constants τ_*n*_ (τ_*n*+1_ > τ_*n*_), to yield a final spectrum, which recovers back to the ground state with time constant τ_fin_. The resulting 2D evolution-associated difference spectra (EADS) are reported in Figure 3b–e, while their integrals over  are reported in Figure 3f. Four components of 0.18, 1.4, 11, (τ_1–3_) and 420 ps (τ_fin_, fixed) were necessary to obtain good fits to the experimental data. The quality of the fit is shown by comparison of experimental and fit traces at selected - pairs (Figure S6).

The first EADS (Figure 3b and blue spectrum in Figure 3f) shows a well-resolved |+⟩, |−⟩ GSB cross-peak and ESA transitions from the FC region of the excited state potential. The spectrum formed in less than 200 fs (Figure 3c) leaves substantially unchanged the high energy ESA. The new feature of this component is the formation of a very weak (∼5% of the GSB) broad positive SE band peaking at  = 15,000 cm^–1^. This is more evident in the normalized 2D EADS integral over the excitation axis, showing the appearance of a positive signal on the red side of the low-energy ESA (green, Figure 3f). Further, saturated 2D EAD spectra are reported in Supporting Information Figure S7.

This weak but reproducible SE feature, which was not resolved in lower time resolution TA measurements,^23,36^ may arise either from fast evolution from the FC state to a more radiative intermediate or from a fast blue shift in the ESA. The former mechanism seems less likely as the states which might be formed (excimer, charge-separated) are expected to have a reduced TDM to S_0_ compared to the FC state. The ESA at  16,700 cm^–1^ also shifts to the blue on the same time scale, which is more consistent with the latter explanation. The fast time scale of the spectral shift likely arises from the initial librational (nondiffusive) component of the DMF solvation time correlation functions, reported as 217 fs by Maroncelli et al. in time-resolved fluorescence upconversion.^37^ Thus, this solvent libration stabilizes the excited state population, hence increasing the S_*n*_ ← S_1_ energy gap and revealing the SE.

The 1.4 ps component (Figure 3d and orange in Figure 3f) shows quenching of the SE feature, and a rise and red-shift of the negative transient feature at  = 20,000 cm^–1^, assigned to SubPc^•–^ absorption, which was previously observed at this wavenumber.^5,15^ Further, redshift of the negative signal at  = 16,700 cm^–1^ is observed. This is due to the decay of the negative FC ESA and simultaneous rise of the negative and red-shifted SubPc^•+^ product absorption. Both the SE decay and the negative band rise time and redshift suggest an initial evolution toward the (dark) SB-CS region of the excited state potential. This component may arise from nuclear relaxation of the inhomogeneously broadened S_1_ population of μ-OSubPc_2_, as suggested by its time scale being close to the 2.2 ps structural evolution component obtained by fsTA for excimer formation in toluene. Inhomogeneous broadening explains the lack of this component from the fsTA data in DMF. In fsTA, the ∼200 cm^–1^ fwhm pump pulses excite a narrow distribution of rotamers relaxing exponentially toward the symmetry-broken state in the polar medium. Conversely, the broad (>1000 cm^–1^ fwhm) excitation pulses used in HB2DES span the vertical transition energies of a wide conformational range of dimers (see Figure 1e). Narrowing of this angular distribution toward formation of the SB-CS state can give rise to this extra component in the dynamics.

The 11 ps component (Figure 3e and solid red in Figure 3f) is dominated by the final formation of the charge separated state, as indicated by the strong product absorption rise time and redshift at 20,800 cm^–1^ and broadened weaker negative band extending to  14,000 cm^–1^. The formation of the final symmetry-broken state is driven by the diffusive response of DMF solvent as previously discussed.^5^ Both the shape and time scale of this EADS are in excellent agreement with fsTA data. The decay time of this component was fixed to 420 ps to match the charge-recombination time leading to the refilling of the ground state observed by fsTA spectroscopy.^5^

The SB-CS dynamics of μ-OSubPc_2_ in DMF were previously reported to occur in a single step driven by diffusive solvent dynamics with a time constant of 11 ps. This is reproduced by HB2DES, but further details of the excited state dynamics of SB-CS are uncovered. Namely, an initial sub-200 fs step relaxes the FC state ESA, causing it to reveal a weak SE. On the 1.4 ps time scale, structural relaxation of the inhomogeneously broadened photoexcited population forms an intermediate state, which already shows a partial CS character, which is followed by stabilization of the full SB-CS state occurring with an 11 ps time constant due to diffusive solvent dynamics. The photoinduced population dynamics of the μ-OSubPc_2_in DMF are summarized by the PES diagram in Figure 3g.

### Wave Packet Dynamics

Given the ultrafast initial evolution of the HB2DES spectra, a role for vibrational wave packets in the excited-state dynamics could be significant. Thus, vibrationally coherent dynamics in SubPc-Cl and μ-OSubPc_2_ were recovered by scanning *T* in 10 fs steps between 0 and 1200 fs to obtain vibrational frequencies and “beatmaps” of resonance Raman active molecular vibrations coupled to the excitonic transitions. Beatmaps are a full frequency domain representation in which the coherent response due to a specific vibration is resolved as a function of the excitation and detection dimensions of the 2D spectrum. These are obtained by a procedure previously discussed in the literature and described in the Supporting Information, Figure S8.^23,38,39^ Our experimental implementation of HB2DES separates the rephasing (photon-echo) and non-rephasing contributions to the 2DES signal based on their evolution in Liouville-space. In the following, we focus on the positive quadrant of the rephasing beatmaps, corresponding to density matrices of the type |*s*_*n*_⟩⟨*s*_*n*+1_| during *T*, where *s* is a generic electronic state and the subscripts indicate vibrational quanta of a generic resonance Raman active mode. Hence, rephasing positive beatmaps are reporting on both ground and excited state vibrational coherences.^23,38^

**Figure 4:**
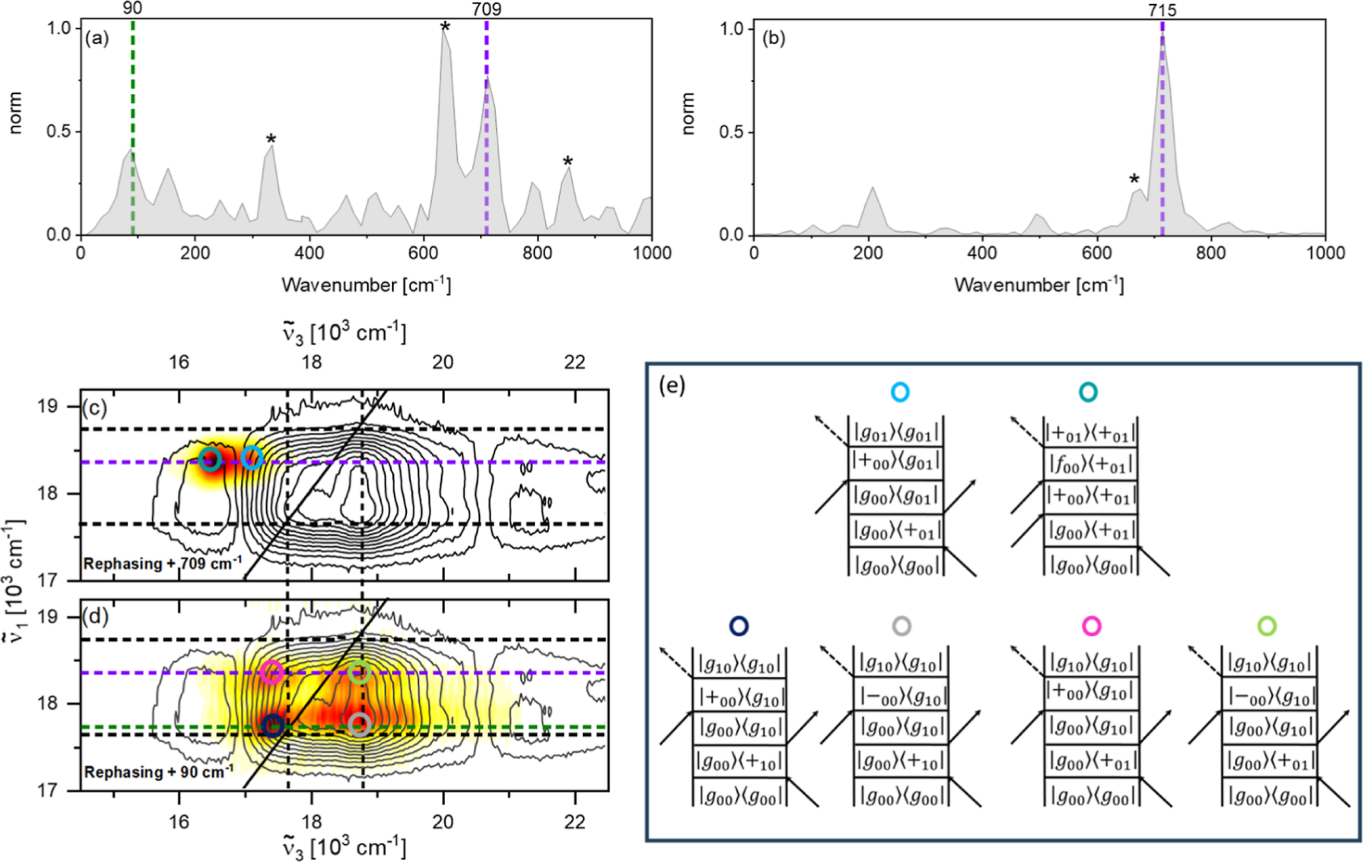
Integrated rephasing vibrational spectra of μ-OSubPc_2_ (a) and SubPc-Cl (b) in DMF. Purple and green dashes in (a) indicate the +709 and +90 cm^–1^ modes, respectively. Purple dash in (b) indicates the +715 cm^–1^ mode. (c, d) Rephasing beatmaps of the +709 and +90 cm^–1^ modes of μ-OSubPc_2_ are shown as white-yellow-red heat maps and amplitude normalized to 1. Black dashes indicate the position of the |+⟩ and |−⟩ excitonic states, purple and green dashes indicate the Raman modes highlighted in (a). (e) Rephasing positive double-sided Feynman diagrams (DSFDs) contributing to the signals observed in the μ-OSubPc_2_ beatmaps in (c,d). Colored circles in the beatmaps mark the DSFDs contributing at a given excitation-detection frequency pair. In the DSFDs, *g*, *+*, *-*, and *f* indicate ground, in-phase exciton, out-of-phase exciton, and a higher excited electronic state, respectively. The first and second digits of the subscripts indicate quanta of vibrational excitation for the +90 and the +709 cm^–1^ modes, respectively.

Selection of the relevant vibrational wavenumbers for beatmap analysis is done from analysis of rephasing vibrational spectra obtained by integration of the 3D spectrum over  and  as described in the Supporting Information. The positive side of the integrated rephasing vibrational spectrum of μ-OSubPc_2_ is reported in Figure 4a. Its most intense features appear at 90 (green dashed line), 156 and 709 (violet dashed line) cm^–1^, with peaks at 323, 670, and 890 cm^–1^ arising from the nonresonant Raman response of the DMF solvent (marked by asterisks). The integrated rephasing vibrational spectrum of SubPc-Cl is shown in Figure 4b and shows an intense signal at 715 cm^–1^ (violet dash) with weaker signals at 200 and 505 cm^–1^ plus a DMF contribution at 670 cm^–1^ (The weaker DMF modes at 323 and 890 cm^–1^ are not resolved on this amplitude scale). The absence of strong signals below 200 cm^–1^ in the integrated and in the calculated vibrational Raman spectra of SubPc-Cl (see Figure S11) suggests assignment of the +90 and +156 cm^–1^ signals to dimer specific modes. Based on DFT calculations and literature,^40^ we assign the strong 709/715 cm^–1^ mode seen in dimer/monomer to an out-of-plane N–C–N bending of the SubPc moiety (see Figure S9). DFT calculations show that the dimer low-frequency modes at 90 and 156 cm^–1^ have much weaker Raman cross sections and correspond to a distortion of the SubPc rings coupled to a B–O–B bending (see Figures S10 and S11). The rephasing beatmaps of the +709 and +90 cm^–1^ dimer modes are shown in Figure 4c, while the rephasing +715 cm^–1^ beatmap of SubPc-Cl is shown in the Supporting Information, Figure S12. As the noise floor of the integrated vibrational spectrum of μ-OSubPc_2_ is ∼0.15 (see baseline of Figure 4a vs b), we test the robustness of the vibronically coherent data by reporting beatmaps at “nonresonant” frequencies i.e. + 60 and 200 cm^–1^ (see Supporting Information, Figure S13). These show that the noise distribution is uncorrelated to molecular resonances and does not produce any “pattern”, which could be misinterpreted for genuine vibrationally coherent signals. Furthermore, the noise amplitude which results from these “off resonance” signals is at least 1 order of magnitude weaker than the beatmaps obtained at resonant Raman frequencies (709 and 90 cm^–1^). Thus, even though these are weak features, they are clearly of molecular origin.

The double-sided Feynman diagrams (DSFDs) representing the Liouville-space pathways of such signals are shown in Figure 4e. In each DSFD, *g*, +, –, and *f* indicate ground, red-shifted exciton, blue-shifted exciton, and a higher excited electronic state, respectively. Quanta of vibrational excitation of the 90 and 709 cm^–1^ modes are indicated by the first and the second digits of the subscripts, respectively.

The rephasing beatmap of the +709 cm^–1^ mode in μ-OSubPc_2_ is shown in Figure 4c while its rephasing negative beatmap is reported in the Supporting Information Figure S15. Along  its amplitude is localized one quantum of vibrational energy to the blue of the |+_00_⟩ band at 17,760 cm^–1^, as highlighted by the purple dash line, consistent with predictions of the displaced harmonic oscillator (DHO) model.^38,41,42^ The position of this feature along  is consistent with S_0_ and S_1_ coherences due to GSB and ESA (light blue and teal circles) signals, respectively. A similar beatmap is retrieved for the rephasing +715 cm^–1^ mode of SubPc-Cl and is shown in the Supporting Information (Figure S12). Our assignment is supported by the rephasing negative 709 cm^–1^ beatmap (which does not report on S_0_ coherences) shown in Figure S15.^38,43,44^

The rephasing +90 cm^–1^ beatmap shows a more complicated pattern comprising four peaks. We note here that all peaks in this low frequency beatmap are broadened as a result of non-negligible thermal population of  = 1 and  = 2 levels within the GS PES (kT at room temperature is ∼200 cm^–1^), which increases the total number of available Liouville-space pathways contributing to the coherent response. The peak at  17,750 cm^–1^ (green dash),  17,400 cm^–1^, marked by a dark blue circle, is assigned to a GSB coherence at +90 cm^–1^ as predicted by the DHO model, as shown in the corresponding DSFD (dark blue circle). This feature is a low-frequency analogue of the GSB signal in the rephasing +709 cm^–1^ beatmap. A weaker feature, not predicted by the DHO and marked by a magenta circle, appears at  17,400 cm^–1^ and  18,300 cm^–1^. As this signal is upshifted from |+_00_⟩ by +709 cm^–1^ along  (purple dash), but appears in the rephasing beatmap “sliced” at +90 cm^–1^, we assign it to a 90 cm^–1^ S_0_ (GSB) coherence, initiated via the first field–matter interaction accessing one quantum of vibrational excitation in the high-frequency (709 cm^–1^) manifold |+_01_⟩, followed by a second interaction stimulating a transition to the  = 1 state of the low-frequency (90 cm^–1^) manifold |*g*_10_⟩. Such a pathway requires coupling between the 709 and 90 cm^–1^ modes, as shown by the DSFD marked by a magenta circle. Anharmonic and harmonic coupling between high- and low-frequency Raman active modes were previously reported in (HB)2DES studies of organic chromophores and in time-resolved Raman studies of diarylethene photoswitches.^4,45−47^

While the signals discussed so far could, in principle, contribute to the coherent response of an uncoupled (monomeric) chromophore, the pair of above diagonal signals, centered at  18,800 cm^–1^ (|−_00_⟩) are unique features of a dimer with multiple bright excitonic states. The signal at  17,750 cm^–1^, marked by a gray circle, is a “replica” of the GSB + 90 cm^–1^ coherence signal marked in dark blue, but probed via a transition to the |−_00_⟩ state, as shown by comparison of their corresponding DSFDs (dark blue and gray circles). Finally, the cross-peak at  18,400 cm^–1^, (light green circle) is assigned to the combined effect of anharmonic coupling and excitation to |+_00_⟩ and detection at |−_00_⟩ wavenumbers, as depicted in the DSFD marked by a light green circle.

The lack of features above the noise floor in the rephasing beatmap obtained at −90 cm^–1^ supports the assignment of these signals to ground state modes. Similarly, no signals at 90 cm^–1^ are detected in the positive non-rephasing data as this part of the non-rephasing response only reports on excited state coherence. The non-rephasing −90 cm^–1^ beatmap is shown in the Supporting Information (Figure S14) and is in good agreement with the rephasing +90 cm^–1^ shown in Figure 4d.

Thus, we have observed an amplitude distribution in a low-frequency rephasing beatmap that is not supported by the DHO model. Such nontrivial signals were explained considering ground state vibrational coherence at 90 cm^–1^ initiated by vibrational coupling to a high frequency (709 cm^–1^) resonance Raman active mode. Further, features specific to an excitonic dimer with multiple bright states are detected in the “above diagonal” region of the rephasing +90 cm^–1^ (and non-rephasing −90 cm^–1^) beatmap and assigned to GS coherence. Hence, the 90 cm^–1^ vibration cannot play any role along the excited state reaction coordinate from the FC to SB-CS, whose main driving force is solvation.

The same argument does not hold for the 709 cm^–1^ vibrational coherence, as beatmap analysis proves it is active in both the ground and excited states, as shown by the teal and blue DSFDs in Figure 4e and by the rephasing −709 cm^–1^ beatmap, which selectively reports on S_1_ coherence (Supporting Information, Figure S15). The rephasing +709 cm^–1^ dimer beatmap and the +715 cm^–1^ beatmap of monomeric SubPc-Cl (Figure S12) are very similar and in excellent agreement with the predictions of a three-level DHO.^38^ Further, both frequencies correspond to a localized out-of-plane bending of the SubPc core not likely to lower the dimer symmetry, as shown by DFT results in Figures S9 and S11. These observations suggest that this excited state vibration is not involved in the formation of the symmetry broken state. A further proof of our assignment of the 709 cm^–1^ SubPc core bending mode to a spectator mode lies in the width of its integrated coherent vibrational response (Figure 4a), inversely proportional to its vibrational dephasing time, being in close agreement with the width of the 715 cm^–1^ mode in SubPc-Cl (Figure 4b). This observation suggests that neither the ultrafast solvation dynamics nor the picosecond structural evolution, both coupled to reaction coordinate, accelerates the dephasing rate of the vibrational wave packet, consistent with its assignment.

Ruling out “spectator” nuclear wave packets is of particular importance for reactive systems, in which a few vibrational modes active along the reaction coordinate could affect photochemistry, while ground and most excited state vibrations are not influencing the reaction rate.

## Conclusions

To conclude, we used HB2DES to uncover fine details on the ultrafast SB-CS of μ-OSubPc_2_ in highly polar DMF solvent. 2D spectra and TD-DFT calculations elucidated the dimer excitonic structure, while 2D global analysis on high signal-to-noise ratio data allowed us to observe subtle features of an intermediate stabilized by the inertial part of the solvent response, and structural relaxation of the inhomogeneously broadened S_1_ population preceding full charge separation. Wave packet dynamics over the first picosecond allowed us to assign low-frequency Raman modes specific to the μ-OSubPc_2_ dimer and vibrational coupling between high- and low-frequency Raman active modes. Furthermore, beatmaps specific to excitonic dimers with a pair of bright states are reported and discussed.

## Supporting Information Available

TD-DFT spectrum and NTOs of SubPc-Cl, instrument response function, additional 2DES data for SubPc-Cl and μ-OSubPc_2_, additional global fit data, scheme of the beatmap calculation method, DFT ground state Raman spectra of SubPc-Cl and μ-OSubPc_2_, displacement vectors of the 709, 715, and 90 cm^–1^ modes, SubPc-Cl rephasing +715 cm^–1^ beatmap, μ-OSubPc_2_ beatmaps at −90 cm^–1^ (nonrephasing) and −709 cm^–1^(rephasing), “nonresonant” frequencies, and optimized S_0_ coordinates of SubPc-Cl and μ-OSubPc_2_ (PDF)
