# Supplementary material for: Symmetry-Breaking Charge-Separation in a Subphthalocyanine Dimer Resolved by Two-Dimensional Electronic Spectroscopy
Source: J Phys Chem C Nanomater Interfaces. 2025 Jan 3;129(2):1069–77. doi: 10.1021/acs.jpcc.4c07588 (PMC11744789; doi:10.1021/acs.jpcc.4c07588)
Supplement: Supplementary file 1 — jp4c07588_si_001.pdf [file jp4c07588_si_001.pdf]

SUPPORTING INFORMATION

Symmetry-Breaking Charge-Separation in a Subphthalocyanine Dimer Resolved by Two-Dimensional Electronic Spectroscopy

Giovanni Bressan*, Isabelle Chambrier, Andrew N. Cammidge and Stephen R. Meech*

School of Chemistry, University of East Anglia, Norwich NR4 7TJ, United Kingdom

Table of Contents:

- TD-DFT spectra and NTOs of SubPc-Cl in DMF p2
- Instrument response function of the HB2DES setup p3
- Absorptive HB2DES of SubPC-Cl in DMF at *T* = 0.2 ps p4
- GSB evolution of the absorptive HB2DES of SubPc dimer in DMF at *T*s between 0 and 125 fs p5
- Integrals over the excitation axis of the absorptive HB2DES of SubPc dimer in DMF p6
- Comparison of absorptive HB2DES traces and global fit of SubPc dimer in DMF p7
- Saturated 2D-EADS highlighting ultrafast SE formation and decay p8
- Summary of the procedure to obtain complex-valued 2D beatmaps p9
- DFT calculations of the 710-715 cm^{-1} modes of SubPc-Cl and SubPc dimer p10
- DFT calculations of the 90 cm^{-1} mode of SubPc dimer p11
- DFT Stick Raman spectra of SubPc-Cl and SubPc dimer p12
- Rephasing +715 cm^{-1} beatmap of SubPc-cl in DMF p13
- Rephasing positive beatmaps of SubPc dimer at “nonresonant” Raman frequencies p14
- Nonrephasing -90 cm^{-1} beatmap of SubPc dimer in DMF p15
- Rephasing -709 cm^{-1} beatmap of SubPc dimer in DMF p16
- Coordinates of SubPc-Cl p17
- Coordinates of SubPc dimer p19
- References p22

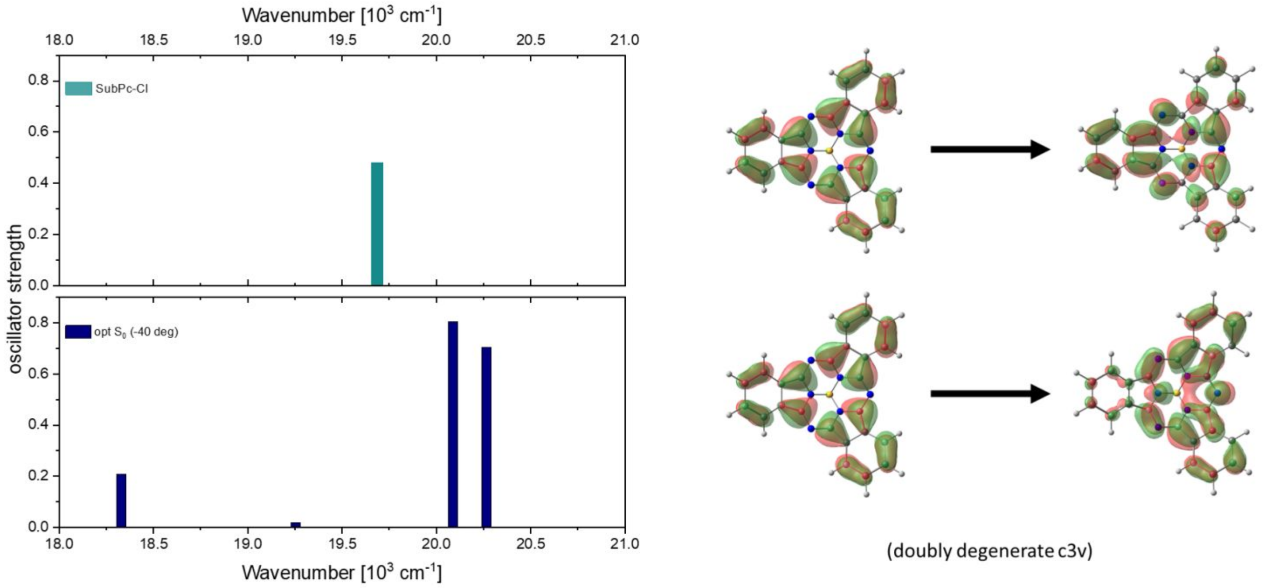

Figure S 1 TD-DFT electronic spectrum of SubPC-Cl (teal) and $SubPc_2O$ (blue) in a PCM model accounting for DMF solvent. Natural Transition Orbital plots of the pair of degenerate transitions of SubPc-Cl. All data shown in this figure was calculated in Gaussian 16.[1]

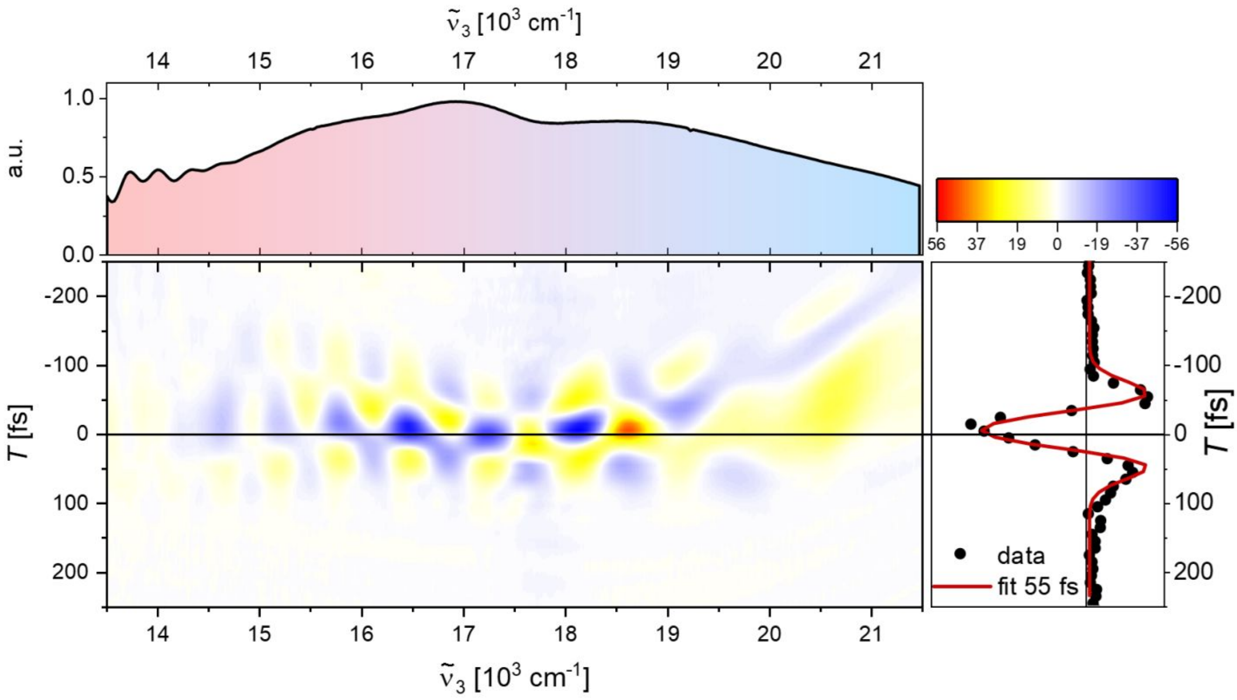

Figure S 2 Instrument response function of the HB2DES setup measured by spectrally resolving the cross correlation of the NOPA pump and the broadband white light continuum (spectrum shown in the top panel), as recorded in a 1 mm cell filled with toluene. The contour shows residual third order dispersion which is not corrected by the dispersive mirrors. Trace and fit of the IRF giving a value of 55 fs are shown in the right panel.

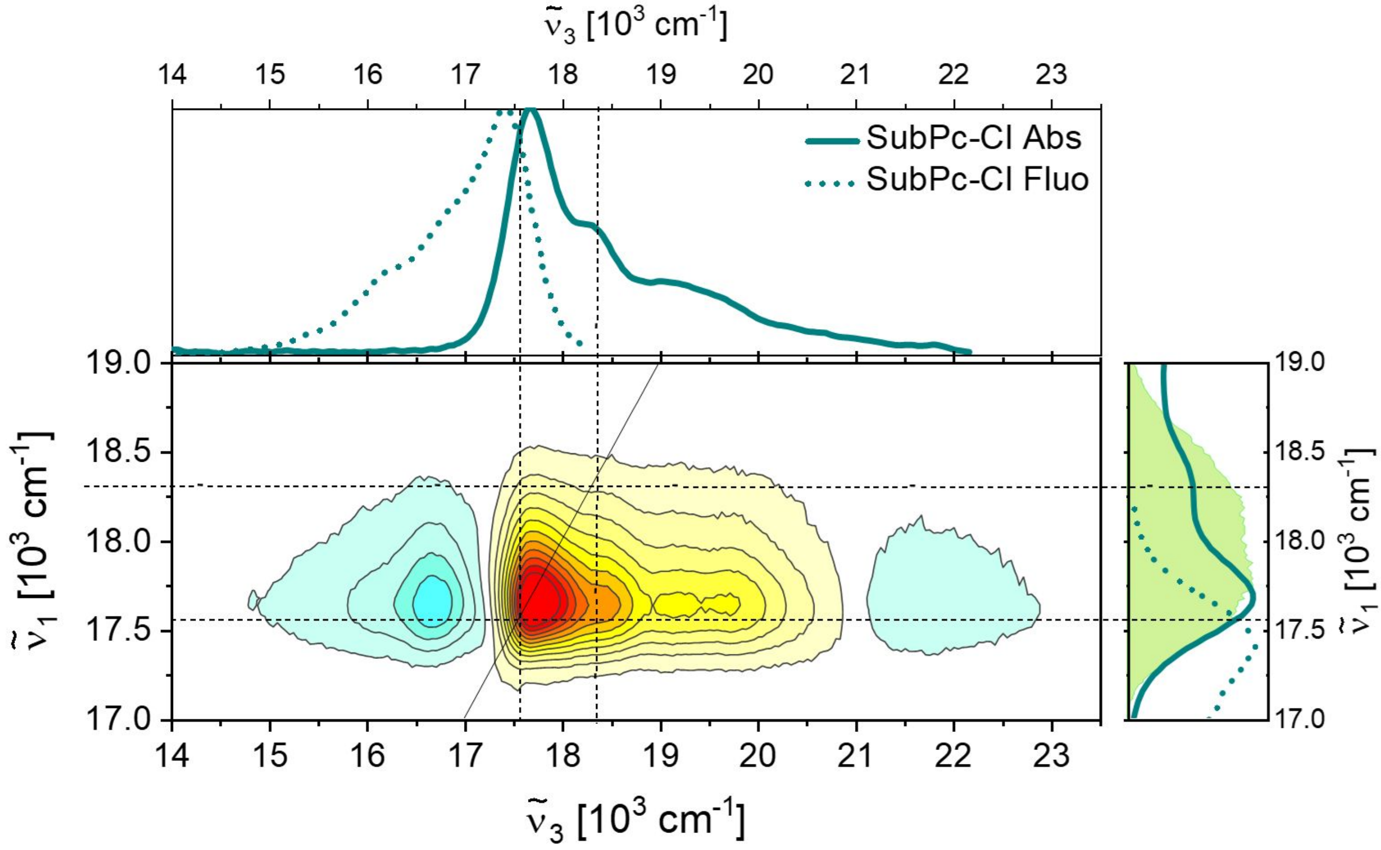

Figure S 3 top panel: normalised steady-state absorption and emission spectra of SubPc-Cl in DMF are reported as solid and dotted teal lines, respectively. These are reproduced, together with the NOPA spectrum shown in shaded green in the right panel. Absorptive HB2DES of SubPc-Cl in DMF at T = 0.2 ps is shown as a 2D contour. The intensity is given by 21 contour lines; positive signals are GSB and SE shown in yellow-orange-red, and negative signals are ESA shown in blue. All spectra are normalized to the positive amplitude. Dashed black lines indicate the 0-0 transition and the most intense vibronic peak of the absorption spectrum.

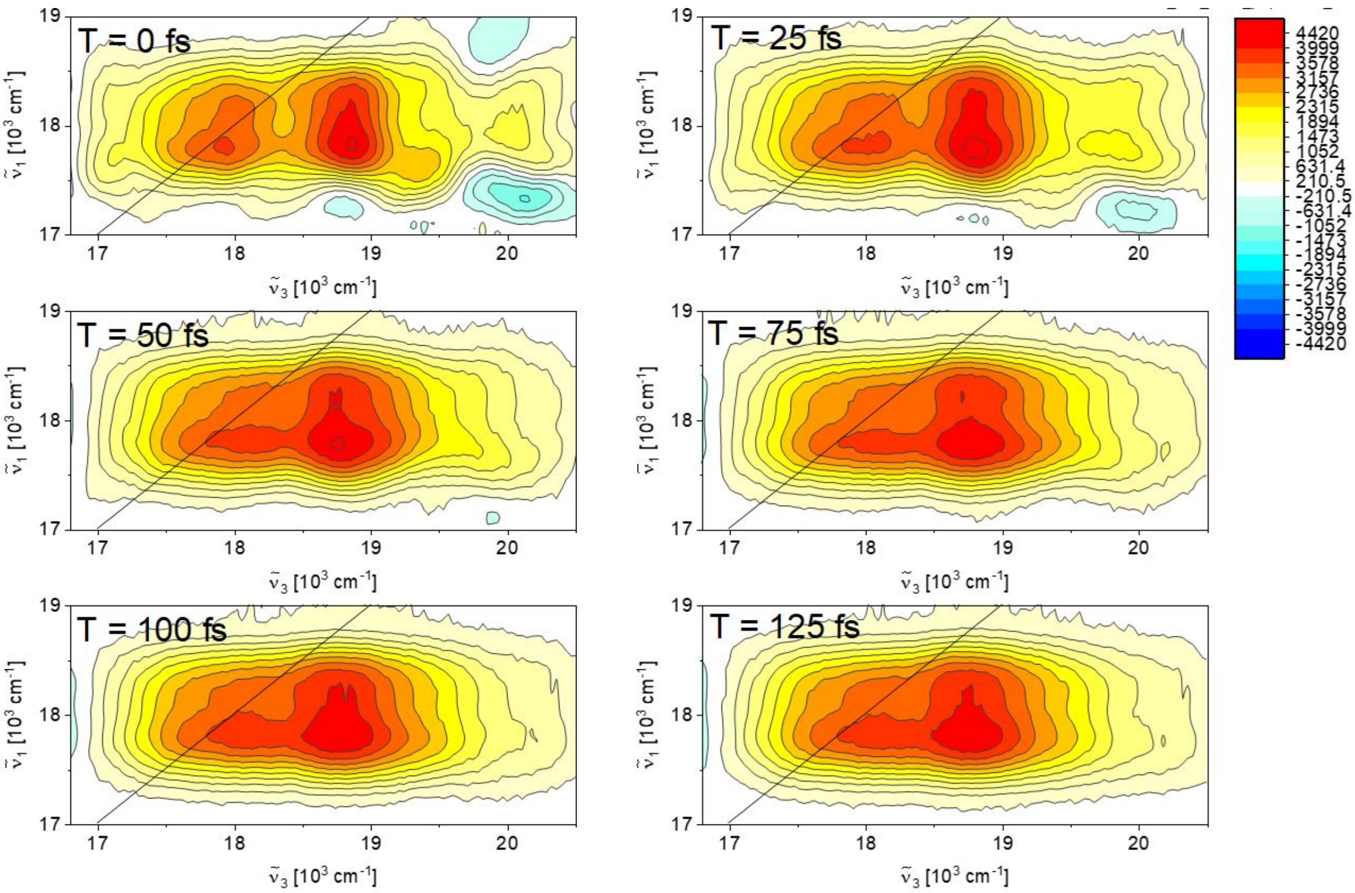

Figure S 4 GSB region of the HB2DES spectra of the SubPc dimer in DMF at *T* values between 0 and 125 fs show the cross peak being present at the earliest times and ultrafast spectral diffusion of the ground-state ensemble. The intensity is given by 21 contour lines; positive signals are shown in yellow-orange-red. All spectra are normalized to the GSB amplitude at *T*= 0 fs.

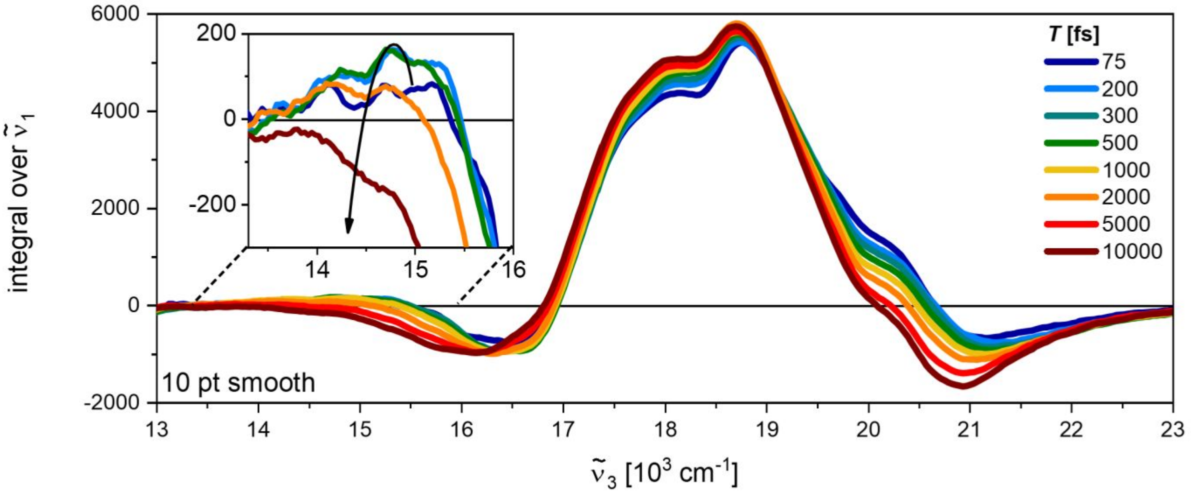

Figure S 5 integral over the excitation axis of the absorptive HB2DES of SubPc dimer in DMF between 75 fs and 10 ps. GSB and SE are positive, and ESA and product absorption are negative. The ultrafast rise and decay of the positive excimer SE feature is ~3% of the strong GSB signal and is better resolved in the inset.

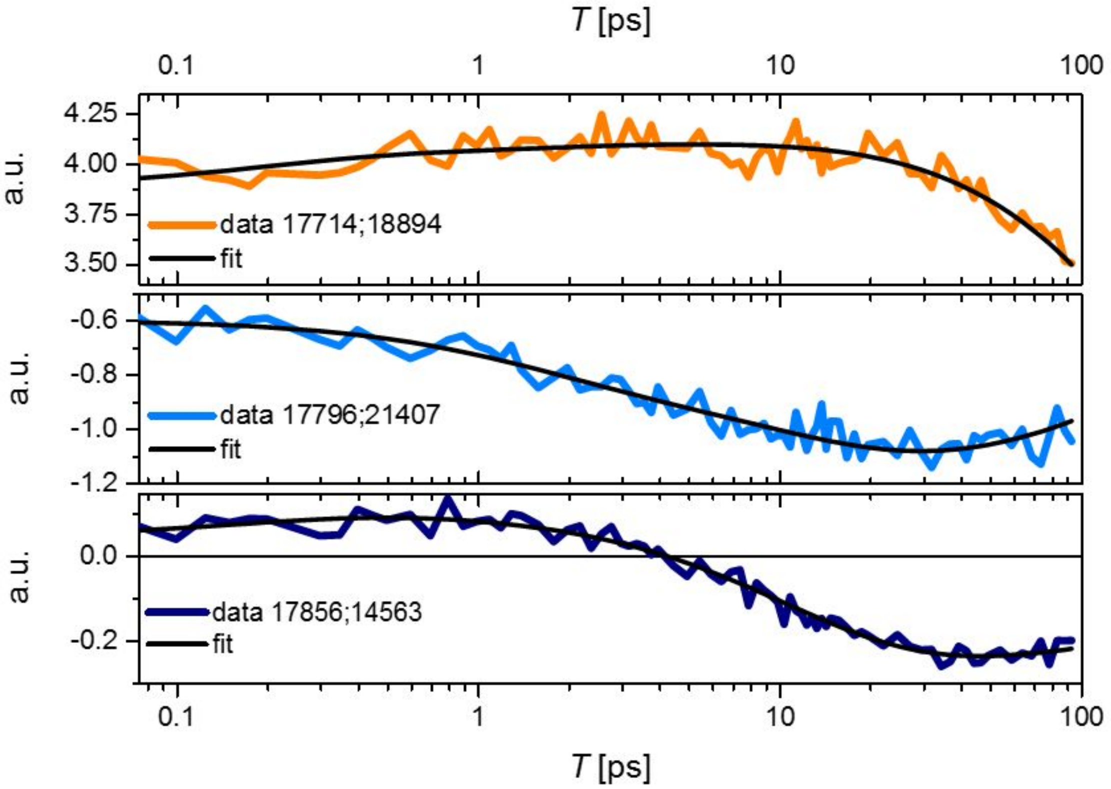

Figure S 6 experimental traces of the absorptive HB2DES data at the GSB, high energy ESA and at the weak SE regions are shown in orange, light and dark blue respectively, overlaid to global fit traces shown in black. Pairs of numbers in the legend indicate excitation and detection frequencies at which every trace was taken.

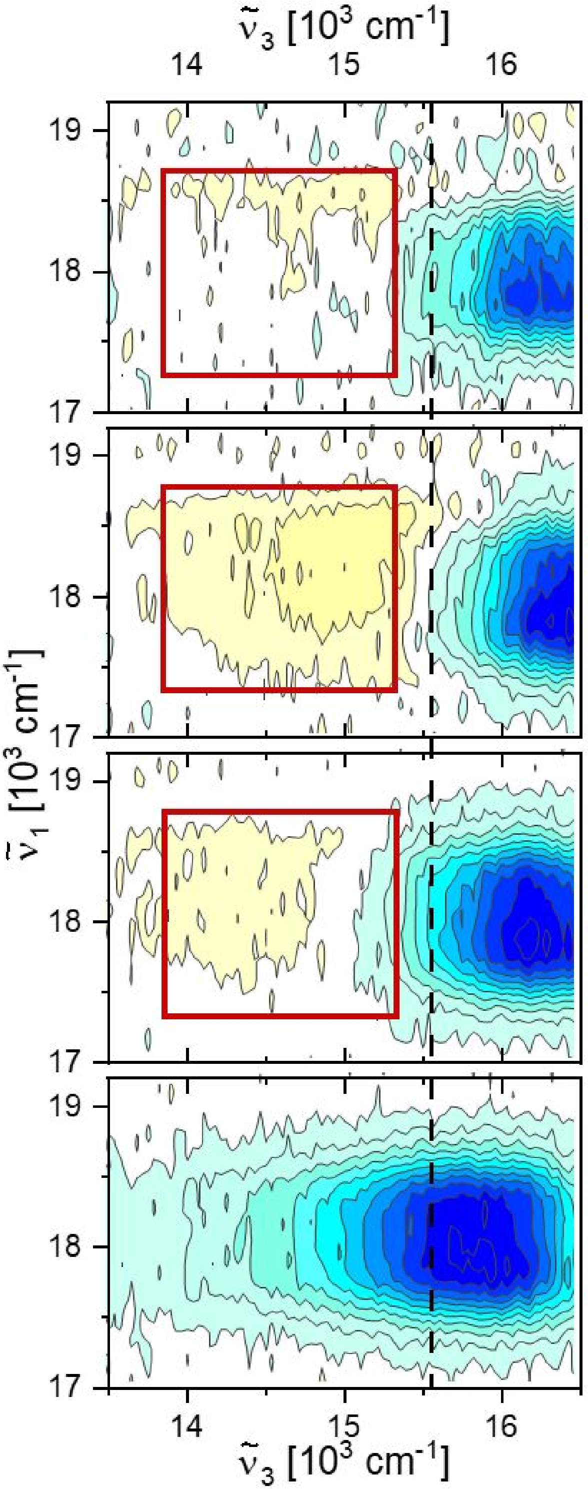

Figure S 7 saturated 2DEADS to highlight the formation of a weak SE feature with a 183fs time constant, which then partially decay and redshifts in 1.4 ps and is fully quenched in 11 ps, when full charge separation occurs. Formation of the SE is highlighted by red squares superimposed to the 2D maps. Further, a vertical dash set at the 0-crossing point between SE and ESA of EADS2 highlights how ultrafast solvation causes an initial ESA blueshift followed by nonexponential redshift and reshaping of the negative peak due to onset of radical anion absorption. The intensity is given by 21 contour lines; positive signals are SE shown in yellow-orange-red, and negative signals are ESA and product absorption and are shown in blue. All spectra are normalized to the 25% of the maximum negative amplitude (ESA/product absorption peaked at 15800 cm^{-1}).

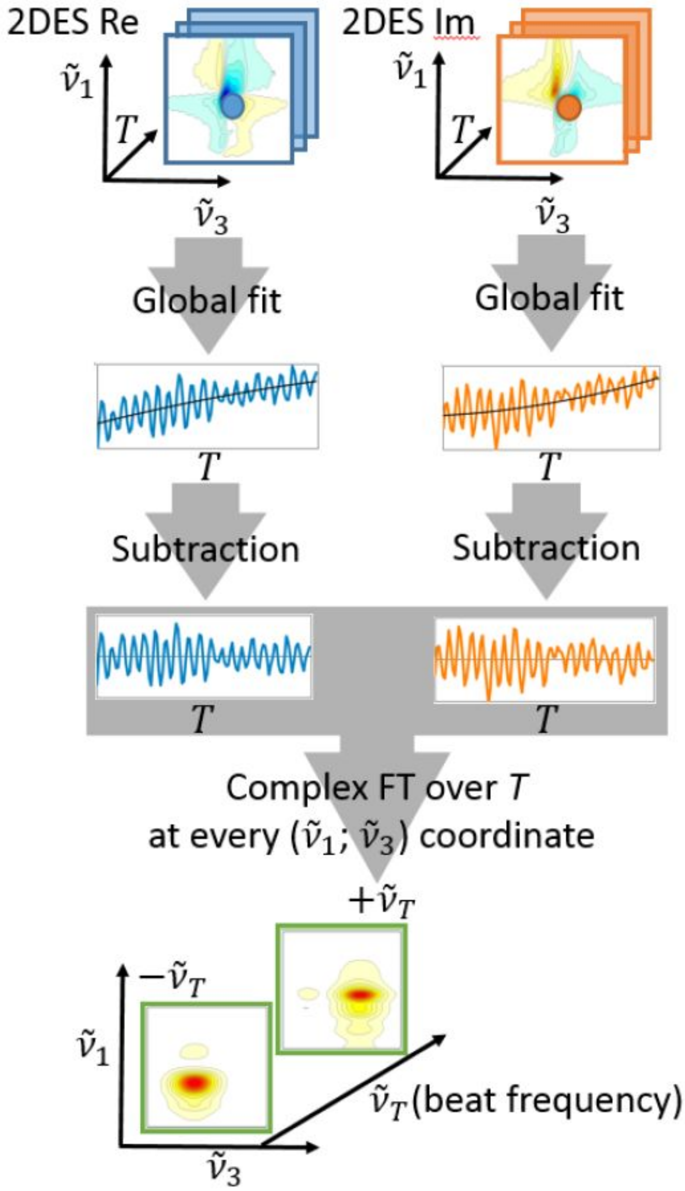

Figure S 8 scheme of the procedure that allows to extract complex valued beatmaps from real and imaginary HB2DES data. Adapted from Bressan et al. [2]

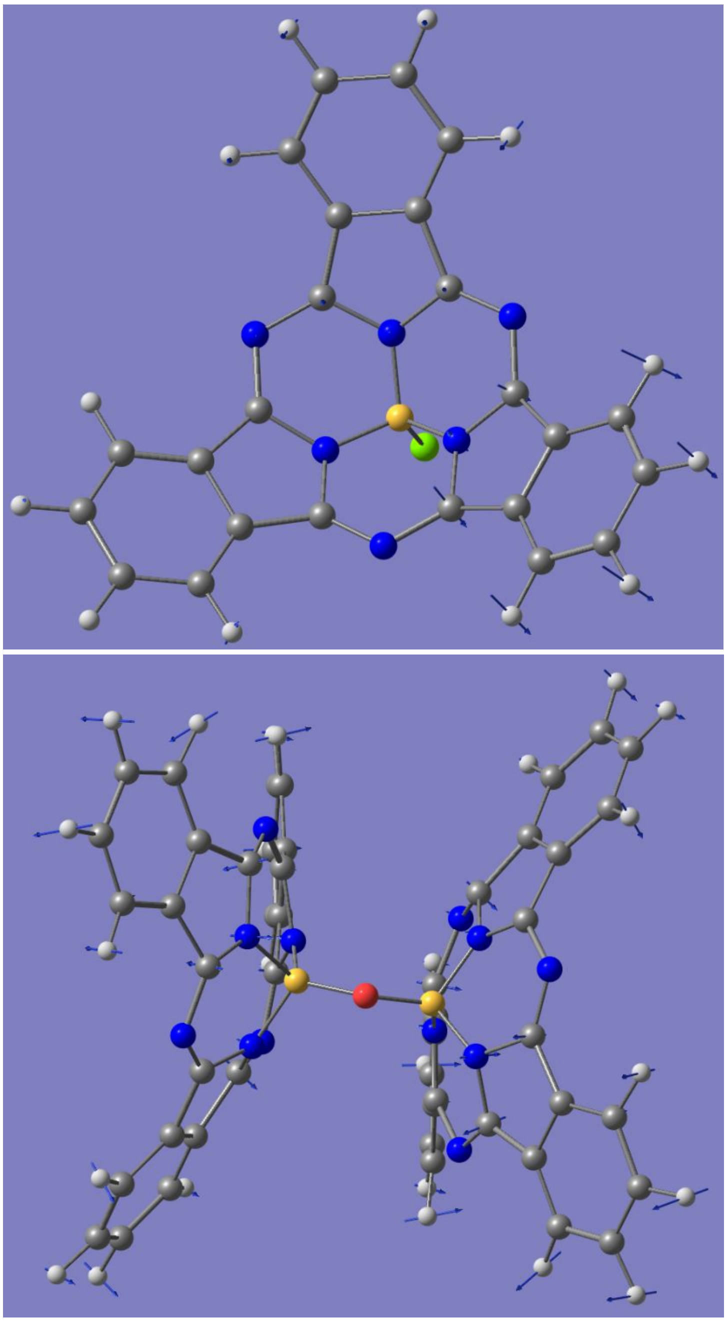

Figure S 9 ground state out-of-plane C-N-C bending of subPc-Cl (top) and SubPc dimer (bottom) at 715/709 cm^{-1} from DFT calculations carried out in Gaussian 16.[1]

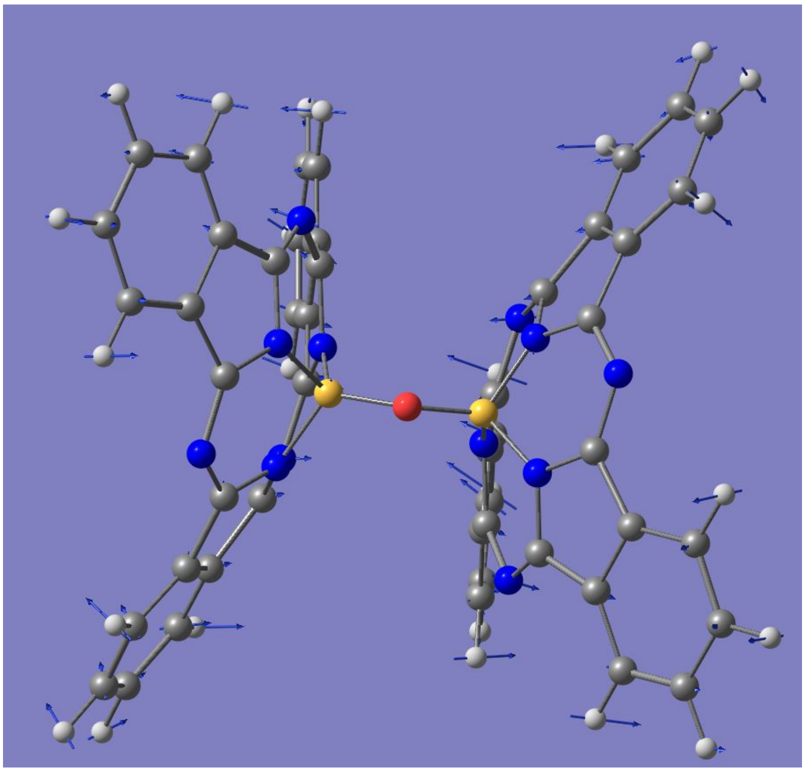

Figure S 10 ground state low frequency torsion around the B-O-B bond of SubPc dimer at 90 cm^{-1} from DFT calculations carried out in Gaussian 16.[1]

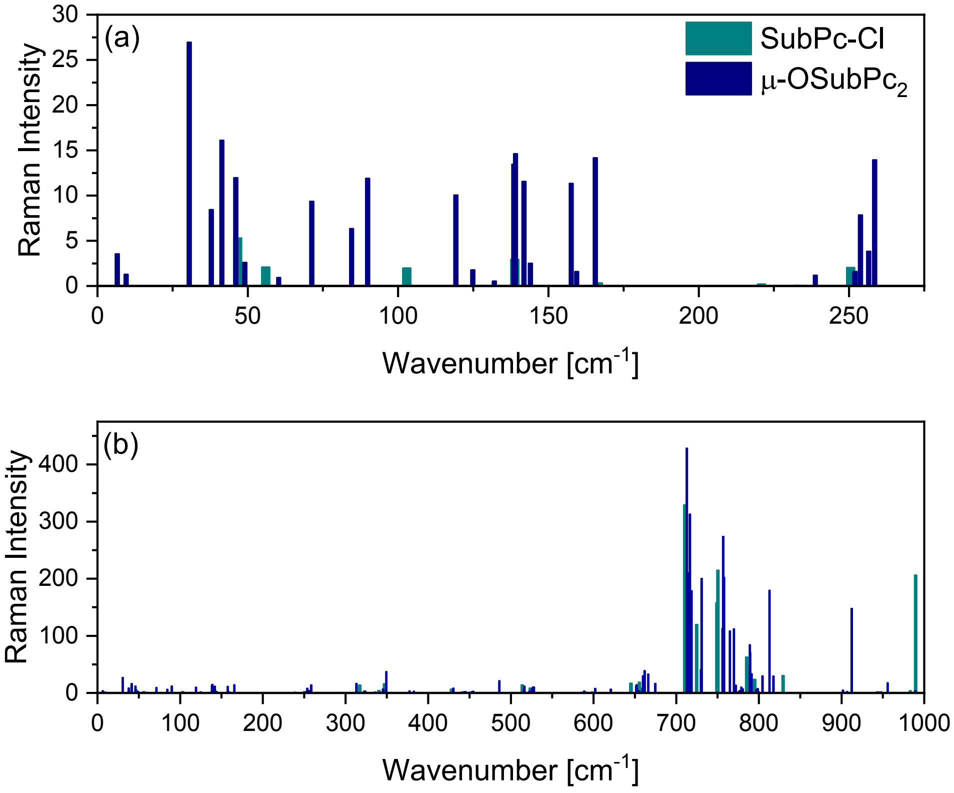

Figure S 11 ground state stick Raman spectra from DFT calculations of SubPc-Cl and SubPc dimer are shown in teal and blue respectively. The low frequency region is reproduced in (a) with a smaller Raman intensity scale. Spectra were calculated in Gaussian 16.[1]

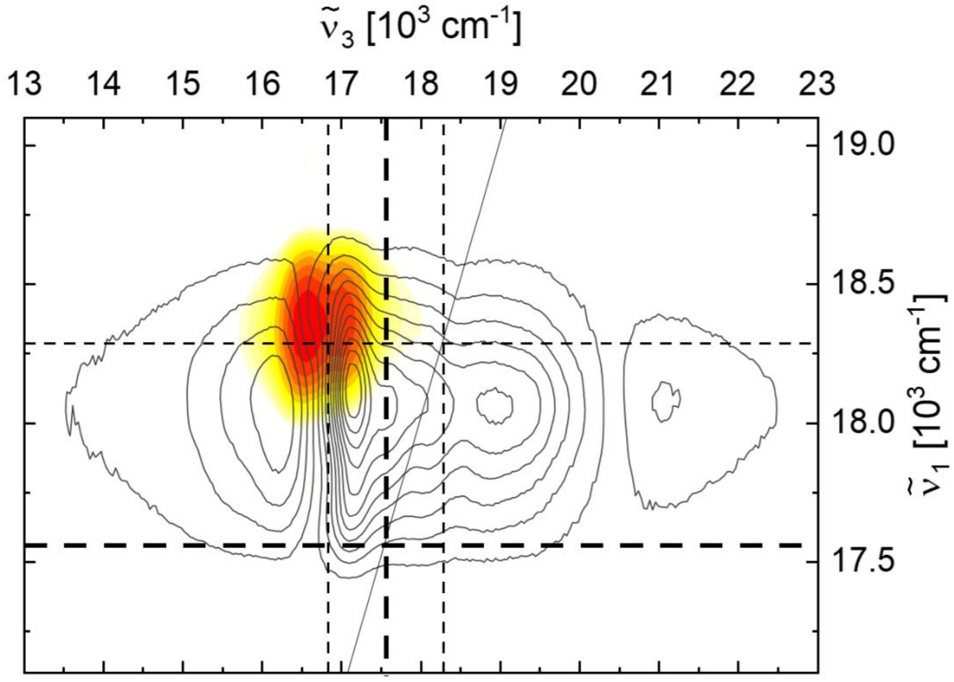

Figure S 12 Rephasing positive beatmap of the 715 cm^{-1} Raman active mode of SubPc-Cl in DMF. Beatmaps are shown as white-yellow-red heatmaps and are all normalised to unity. Contour lines showing the real part of the corresponding absorptive 2D spectrum (*T* = 0.2 fs) are overlaid to the beatmap. Vertical and horizontal thick dashed lines are drawn at the 0-0 electronic transition frequencies, whilst thin lines are at +/- 1 quanta and +1 quantum of vibrational excitation from the pure electronic transitions on the excitation and detection axes, respectively. The beatmap amplitude distribution suggests GSB and overlaid SE and ESA contributions to the coherent response of SubPc-Cl at 715 cm^{-1}, in agreement with a three-level displaced harmonic oscillator (DHO) model.[2]

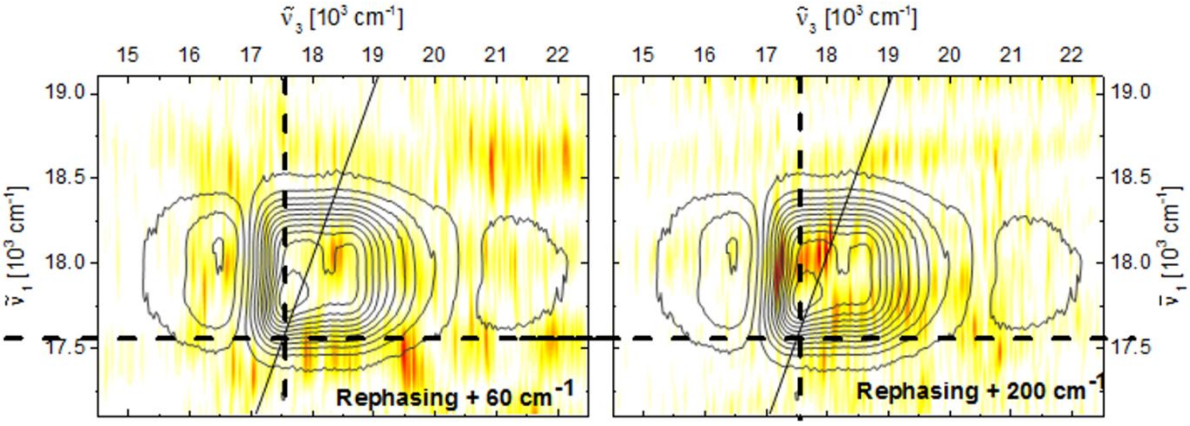

Figure S 13 Rephasing positive beatmaps at 60 and 200 cm-1, where no raman Raman active mode of SubPc dimer are present. Beatmaps are shown as white-yellow-red heatmaps and are all normalised to unity. Contour lines showing the real part of the corresponding absorptive 2D spectra (T = 0.2 fs) are overlaid to the beatmap. Vertical and horizontal thick dashed lines are drawn at the 0-0 electronic transition frequencies.

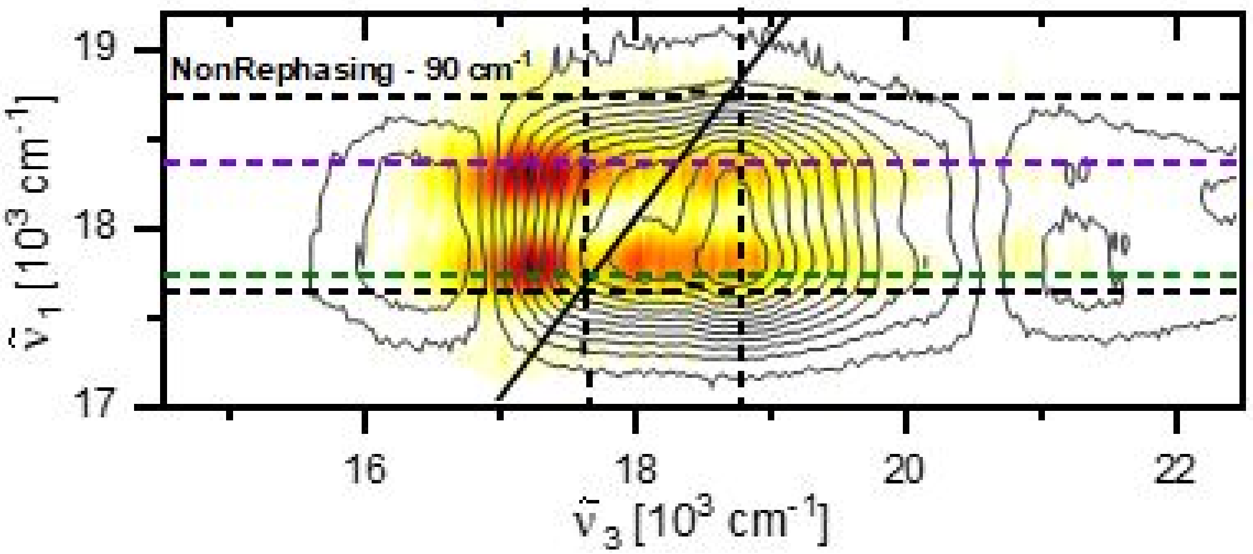

Figure S 14 Nonrephasing negative beatmap of the 90 cm^{-1} Raman active mode of SubPc dimer in DMF. Beatmaps are shown as white-yellow-red heatmaps and are all normalised to unity. Contour lines showing the real part of the corresponding absorptive 2D spectrum (T = 0.2 fs) are overlaid to the beatmap. Vertical and horizontal black dashed lines are drawn at the |+> and |-> electronic transition frequencies, whilst green and purple dashed lines are displaced (along the excitation axis) by the |+> energy level by one quantum of vibrational excitation of the low (90 cm^{-1}) and high frequency (709 cm^{-1}) Raman active modes analysed in the paper. The beatmap amplitude distribution is consistent with the rephasing beatmap at +90 cm^{-1} and shows contributions from the nonrephasing counterparts of the double sided Feynman diagrams shown in Figure 4e and thus assigned to ground state coherence.

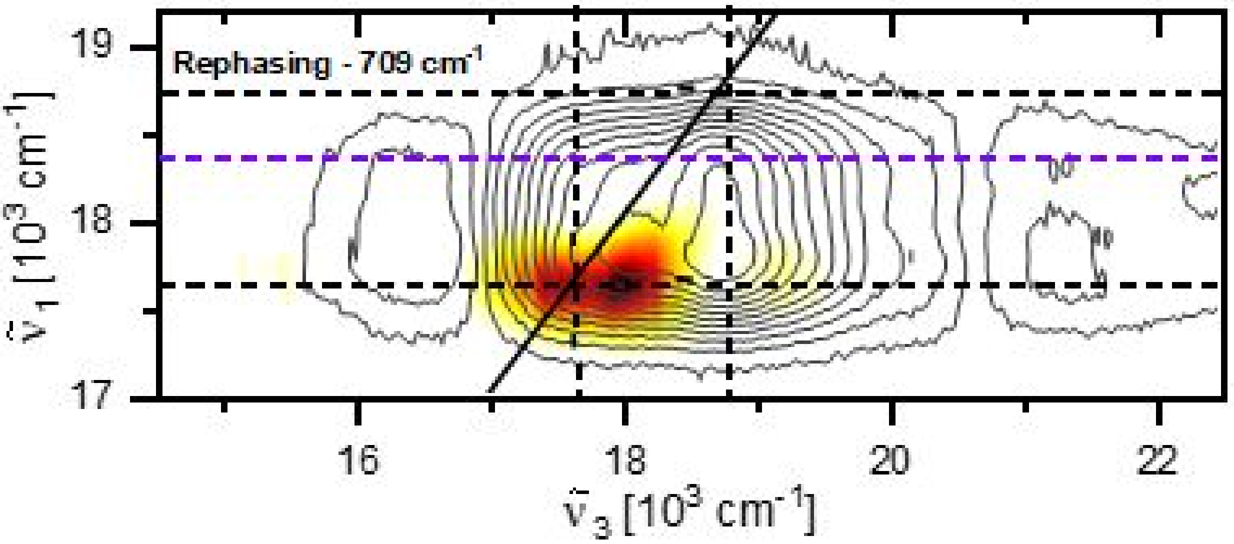

Figure S 15 Rephasing negative beatmap of the 709 cm^{-1} Raman active mode of SubPc dimer in DMF. Beatmaps are shown as white-yellow-red heatmaps and are all normalised to unity. Contour lines showing the real part of the corresponding absorptive 2D spectrum (T = 0.2 fs) are overlaid to the beatmap. Vertical and horizontal black dashed lines are drawn at the |+> and |-> electronic transition frequencies, whilst the purple dash is displaced (along the excitation axis) by the |+> energy level by one quantum of vibrational excitation of the high frequency (709 cm^{-1}) Raman active mode. The beatmap amplitude distribution is consistent with the rephasing beatmap at +90 cm^{-1} and shows contributions from excited state coherence due to either SE or ESA pathways and are one quantum of vibrational excitation lower than the rephasing positive beatmap, according to the DHO model.[2]

-

SubPc-Cl coordinates

Center Number	Atomic Number	Atomic Type	Coordinates (Angstroms) X	Y	Z
1	6	0	-1.130950	2.017193	0.048261
2	7	0	0.000000	1.373164	0.292306
3	6	0	1.130950	2.017193	0.048261
4	6	0	-0.696266	3.472305	-0.233659
5	6	0	0.696266	3.472305	-0.233659
6	6	0	-1.434921	4.624045	-0.466876
7	6	0	-0.713303	5.816794	-0.705100
8	6	0	0.713303	5.816794	-0.705100
9	6	0	1.434921	4.624045	-0.466876
10	7	0	-2.366224	1.366140	0.058806
11	6	0	-2.312415	-0.029165	0.048261
12	7	0	-1.189195	-0.686582	0.292306
13	6	0	-3.355237	-1.133169	-0.233659
14	6	0	-2.658972	-2.339136	-0.233659
15	6	0	-1.181465	-1.988028	0.048261
16	6	0	-4.722001	-1.069345	-0.466876
17	6	0	-5.394143	-2.290659	-0.705100
18	6	0	-4.680840	-3.526135	-0.705100
19	6	0	-3.287080	-3.554700	-0.466876
20	7	0	-0.000000	-2.732280	0.058806
21	6	0	1.181465	-1.988028	0.048261
22	7	0	1.189195	-0.686582	0.292306
23	6	0	2.312415	-0.029165	0.048261
24	6	0	3.355237	-1.133169	-0.233659
25	6	0	2.658972	-2.339136	-0.233659
26	6	0	4.722001	-1.069345	-0.466876

27	6	0	5.394143	-2.290659	-0.705100
28	6	0	4.680840	-3.526135	-0.705100
29	6	0	3.287080	-3.554700	-0.466876
30	7	0	2.366224	1.366140	0.058806
31	5	0	-0.000000	0.000000	0.810254
32	1	0	5.244878	-0.135803	-0.466480
33	1	0	6.448490	-2.287983	-0.887432
34	1	0	5.205696	-4.440565	-0.887432
35	1	0	2.740048	-4.474296	-0.466480
36	1	0	-5.244878	-0.135803	-0.466480
37	1	0	-6.448490	-2.287983	-0.887432
38	1	0	-5.205696	-4.440565	-0.887432
39	1	0	-2.740048	-4.474296	-0.466480
40	1	0	-2.504830	4.610099	-0.466480
41	1	0	2.504830	4.610099	-0.466480
42	1	0	-1.242794	6.728548	-0.887432
43	1	0	1.242794	6.728548	-0.887432
44	17	0	-0.000000	0.000000	2.680254

SubPc dimer coordinates

Center Number	Atomic Number	Atomic Type	X	Y	Z
1	6	0	-1.967316	-1.675088	-1.351129
2	7	0	-1.880122	-1.329751	-0.033543
3	6	0	-2.558934	-2.180423	0.791801
4	6	0	-2.535278	-3.015129	-1.370611
5	6	0	-2.904932	-3.331470	-0.029634
6	6	0	-2.825593	-3.894315	-2.410360
7	6	0	-3.455418	-5.092118	-2.102131
8	6	0	-3.819327	-5.403265	-0.782937
9	6	0	-3.562161	-4.524109	0.259882
10	7	0	-1.837866	-0.780654	-2.336553
11	6	0	-1.777631	0.512034	-2.000978
12	7	0	-1.673813	0.930801	-0.703991
13	6	0	-2.135827	1.694384	-2.768819
14	6	0	-2.384192	2.742062	-1.833321
15	6	0	-2.175922	2.186932	-0.504602
16	6	0	-2.342861	1.898434	-4.130528
17	6	0	-2.767972	3.150750	-4.550597
18	6	0	-3.012818	4.181785	-3.629956
19	6	0	-2.838241	3.984888	-2.267514
20	7	0	-2.650730	2.593800	0.677240
21	6	0	-2.788527	1.677825	1.641375
22	7	0	-2.321955	0.404206	1.509420
23	6	0	-2.980163	-0.500444	2.287139
24	6	0	-3.795137	0.288749	3.200729
25	6	0	-3.675308	1.652060	2.796482
26	6	0	-4.652700	-0.068192	4.237272
27	6	0	-5.361143	0.937358	4.881030

28	6	0	-5.243126	2.277778	4.483724
29	6	0	-4.413810	2.645529	3.432825
30	7	0	-3.036644	-1.801016	1.981444
31	5	0	-1.323942	0.001066	0.444286
32	1	0	-4.756571	-1.104594	4.531468
33	1	0	-6.021231	0.684656	5.701586
34	1	0	-5.814247	3.036457	5.004406
35	1	0	-4.335627	3.676907	3.114035
36	1	0	-2.170740	1.097237	-4.837459
37	1	0	-2.920163	3.337184	-5.606546
38	1	0	-3.349405	5.145460	-3.991849
39	1	0	-3.044291	4.773260	-1.555184
40	1	0	-2.560169	-3.645457	-3.429665
41	1	0	-3.858837	-4.754719	1.274885
42	1	0	-3.676094	-5.798723	-2.892644
43	1	0	-4.314444	-6.344537	-0.578542
44	1	0	5.813525	-3.041070	5.002054
45	1	0	4.334788	-3.679695	3.111157
46	6	0	5.242560	-2.281856	4.481981
47	6	0	4.413175	-2.648591	3.430781
48	1	0	6.020979	-0.689873	5.701132
49	6	0	5.360844	-0.941780	4.880369
50	1	0	3.043275	-4.772058	-1.558946
51	6	0	3.674881	-1.654460	2.795233
52	7	0	2.650137	-2.594292	0.675221
53	1	0	3.348449	-5.142330	-3.995892
54	6	0	2.837433	-3.983056	-2.270641
55	6	0	4.652607	0.064430	4.237417
56	6	0	2.788123	-1.679110	1.640085
57	6	0	3.012046	-4.178878	-3.633232
58	6	0	3.794982	-0.291502	3.200581

59	6	0	2.175434	-2.186377	-0.506299
60	6	0	2.383628	-2.740487	-1.835456
61	1	0	4.756684	1.100576	4.532446
62	7	0	2.321833	-0.405287	1.509126
63	6	0	2.980202	0.498595	2.287600
64	6	0	2.767479	-3.147039	-4.553048
65	7	0	1.673596	-0.929973	-0.704696
66	5	0	1.323922	-0.001082	0.444289
67	6	0	2.135533	-1.691999	-2.770118
68	1	0	2.919697	-3.332643	-5.609139
69	6	0	2.342605	-1.894980	-4.131982
70	7	0	3.036964	1.799401	1.982952
71	6	0	1.777557	-0.510194	-2.001347
72	7	0	1.880406	1.329986	-0.032444
73	6	0	2.559377	2.179857	0.793598
74	1	0	2.170697	-1.093165	-4.838264
75	7	0	1.838088	0.782745	-2.335895
76	6	0	1.967710	1.676357	-1.349749
77	6	0	2.905648	3.331486	-0.026911
78	6	0	2.535968	3.016286	-1.368149
79	1	0	3.859804	4.753514	1.278756
80	6	0	3.563111	4.523764	0.263563
81	6	0	2.826497	3.896228	-2.407197
82	6	0	3.820483	5.403692	-0.778554
83	1	0	2.561061	3.648225	-3.426708
84	6	0	3.456554	5.093660	-2.098005
87	8	0	-0.000010	-0.000026	0.926433

References

(1) Frisch, M. J.; Trucks, G. W.; Schlegel, H. B.; Scuseria, G. E.; Robb, M. a.; Cheeseman, J. R.; Scalmani, G.; Barone, V.; Petersson, G. a.; Nakatsuji, H.; Li, X.; Caricato, M.; Marenich, a. V.; Bloino, J.; Janesko, B. G.; Gomperts, R.; Mennucci, B.; Hratchian, H. P.; Ortiz, J. V.; Izmaylov, a. F.; Sonnenberg, J. L.; Williams; Ding, F.; Lipparini, F.; Egidi, F.; Goings, J.; Peng, B.; Petrone, A.; Henderson, T.; Ranasinghe, D.; Zakrzewski, V. G.; Gao, J.; Rega, N.; Zheng, G.; Liang, W.; Hada, M.; Ehara, M.; Toyota, K.; Fukuda, R.; Hasegawa, J.; Ishida, M.; Nakajima, T.; Honda, Y.; Kitao, O.; Nakai, H.; Vreven, T.; Throssell, K.; Montgomery Jr., J. a.; Peralta, J. E.; Ogliaro, F.; Bearpark, M. J.; Heyd, J. J.; Brothers, E. N.; Kudin, K. N.; Staroverov, V. N.; Keith, T. a.; Kobayashi, R.; Normand, J.; Raghavachari, K.; Rendell, a. P.; Burant, J. C.; Iyengar, S. S.; Tomasi, J.; Cossi, M.; Millam, J. M.; Klene, M.; Adamo, C.; Cammi, R.; Ochterski, J. W.; Martin, R. L.; Morokuma, K.; Farkas, O.; Foresman, J. B.; Fox, D. J. Gaussian16. 2016, p Gaussian 16, Revision C.01, Gaussian, Inc., Wallin.

(2) Bressan, G.; Green, D.; Jones, G. A.; Heisler, I. A.; Meech, S. R. Two-Dimensional Electronic Spectroscopy Resolves Relative Excited-State Displacements. *J Phys Chem Lett* **2024**, *15* (10), 2876–2884. https://doi.org/10.1021/acs.jpclett.3c03420.
